# Age-specific sequence of colorectal cancer screening options in Germany: A model-based critical evaluation

**DOI:** 10.1371/journal.pmed.1003194

**Published:** 2020-07-17

**Authors:** Thomas Heisser, Korbinian Weigl, Michael Hoffmeister, Hermann Brenner

**Affiliations:** 1 Division of Clinical Epidemiology and Aging Research, German Cancer Research Center (DKFZ), Heidelberg, Germany; 2 Medical Faculty Heidelberg, University of Heidelberg, Heidelberg, Germany; 3 German Cancer Consortium (DKTK), German Cancer Research Center (DKFZ), Heidelberg, Germany; 4 Division of Preventive Oncology, German Cancer Research Center (DKFZ) and National Center for Tumor Diseases (NCT), Heidelberg, Germany; University of Melbourne School of Population and Global Health, AUSTRALIA

## Abstract

**Background:**

The current organized screening program for colorectal cancer in Germany offers both sexes 5 annual fecal immunochemical tests (FITs) between ages 50 and 54 years, followed by a first screening colonoscopy at age 55 years if all of these FITs were negative. We sought to assess the implications of this approach for key parameters of diagnostic performance.

**Methods and findings:**

Using a multistate Markov model, we estimated the expected detection rates of advanced neoplasms (advanced adenomas and cancers) and number needed to scope (NNS) to detect 1 advanced neoplasm at a first screening colonoscopy conducted at age 55 after 5 preceding negative FITs and compared them with the corresponding estimates for a first screening colonoscopy at age 55 with no preceding FIT testing. In individuals with 5 consecutive negative FITs undergoing screening colonoscopy at age 55, expected colonoscopy detection rate (NNS) was 3.7% (27) and 0.10% (1,021) for any advanced neoplasm and cancer, respectively, in men, and 2.1% (47) and 0.05% (1,880) for any advanced neoplasm and cancer, respectively, in women. These NNS values for detecting 1 advanced neoplasm are approximately 3-fold higher, and the NNS values for detecting 1 cancer are approximately 8-fold higher, than those for a first screening colonoscopy at age 55 without prior FITs. This study is limited by model simplifying assumptions and uncertainties related to input parameters.

**Conclusions:**

Screening colonoscopy at age 55 after 5 consecutive negative FITs at ages 50–54, as currently offered in the German cancer early detection program, is expected to have very low positive predictive value. Our results may inform efforts to enhance the design of screening programs.

## Abstract 2

Thomas Heisser and colleagues model outcomes from colorectal cancer screening in Germany.

## Author summary

**Why was this study done?:**

- The current screening approach for colorectal cancer (CRC) in Germany offers both men and women 5 annual fecal immunochemical tests (FITs, a test for blood in stool) at ages 50–54, followed by a first screening colonoscopy at age 55 in case all of these FITs were negative.
- The expected result of this sequential screening strategy is unknown.

**What did the researchers do and find?:**

- In a simulated population of 100,000 men and 100,000 women, colonoscopy detection rates for cancer at age 55 were at or below 0.10% for both sexes after 5 consecutive negative FITs.
- Looked at another way, 1,000 colonoscopies would be needed in men and 1,900 colonoscopies would be needed in women to detect 1 CRC. These numbers are 8-fold higher than those for a first screening colonoscopy at age 55 without prior FITs.

**What do these findings mean?:**

- Although annual FITs at ages 50–54 may contribute to earlier diagnosis of CRC, the current sequence of CRC screening, if fully adhered to, may not use screening capacities in the most efficient manner.
- Our results should inform and encourage efforts to design more rational sequences of screening offers.

## Introduction

Colorectal cancer (CRC) is the fourth most commonly diagnosed cancer and the third leading cause of cancer death in Europe, accounting for approximately 60,000 new cancer cases and approximately 25,000 cancer deaths every year in Germany alone [1,2]. The slow progression from adenomatous polyps to invasive cancers, along with the existence of effective methods to detect and remove adenomas, makes screening an effective and cost-effective approach to lower the burden of CRC [3–7]. Screening approaches recommended by international expert panels [8] and offered in screening programs across Europe [9] include colonoscopy, sigmoidoscopy, and fecal immunochemical tests (FITs) for hemoglobin. However, typically just 1 screening approach is offered as the primary screening test. Another screening modality may be offered as a second-tier option for individuals who refuse or are not eligible for the primary test [9].

The screening approach in Germany is unusual in several respects. First, 2 options are offered as the primary screening test: colonoscopy and FIT. Second, in April 2019, different starting ages were introduced for eligibility for screening colonoscopy among men (50 years) and women (55 years). Third, between the ages 50 and 54 years, both sexes are offered annual FIT, followed by either 2 screening colonoscopies 10 years apart or FIT-based screening every 2 years from age 55 onwards (Table 1). With the former, men and women are offered 5 annual FITs at ages 50–54, followed by a first screening colonoscopy at age 55 if all of these FITs were negative. However, having had 5 consecutive negative annual FITs at ages 50–54 is expected to yield a low prevalence of advanced neoplasms at age 55, with associated high numbers of colonoscopies needed to detect 1 advanced adenoma or 1 case of CRC (number needed to scope [NNS]). This might make screening colonoscopy at age 55 rather inefficient.

**Table 1:** Screening approach in Germany.

Screening test	Men	Women
Colonoscopy	Start of eligibility: age 50 years	Start of eligibility: age 55 years
	2 screening colonoscopies 10 years apart if first screening colonoscopy was before age 65	2 screening colonoscopies 10 years apart if first screening colonoscopy was before age 65
Fecal immunochemical test*	Annually from age 50 to 54 if no screening colonoscopy is used	Annually from age 50 to 54
	Biennially from age 55 onwards if no screening colonoscopy is used	Biennially from age 55 onwards if no screening colonoscopy is used

*Following a positive fecal immunochemical test, diagnostic colonoscopy is warranted.

The objective of this modeling study was to assess the implications of this approach for key parameters of diagnostic performance. First, we assessed the detection rate of advanced neoplasms and associated NNS of colonoscopy screening at age 55 in individuals perfectly adhering to the annual FIT screening offered at ages 50–54 and compared these estimates to those calculated for strategies involving no or less intensive preceding FIT screening. Second, we assessed the yearly performance of consecutive annual FIT testing in terms of its positive predictive value (PPV) and negative predictive value (NPV).

## Methods

### Multistate Markov model

**Fig 1:**
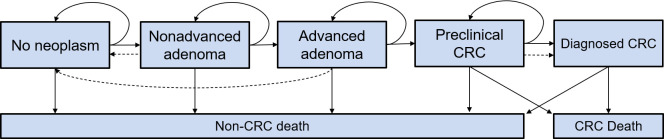
Schematic illustration of the Markov model. Solid arrows represent the progression of colorectal disease through the adenoma–carcinoma sequence in the absence of screening; dashed arrows show the movement between states because of the detection and removal of adenomas and the detection of asymptomatic CRC at screening. CRC, colorectal cancer.

We set up a multistate Markov model to simulate the natural history of CRC based on the adenoma–carcinoma process (Fig 1). Background information on the model’s structure and data sources has been reported previously [10–14]. Briefly, simulation is performed on a hypothetical previously unscreened German population of 100,000 men and 100,000 women for a predefined number of years, whereby screening can interfere with the natural history of CRC. Details are reported in S1 Text, and overviews on the parameters used in the model can be found in S1–S3 Tables.

### Simulations

#### Modeled strategies

We performed a simulation for the current approach of sequential FIT–colonoscopy screening within the organized screening program in Germany, i.e., 5 consecutive annual FITs offered at ages 50–54 years followed by a screening colonoscopy at age 55 years, assuming a perfectly adhering population.

For comparison, we first estimated a model with no FIT screening at ages 50–54, i.e., individuals received only screening colonoscopy at age 55. Second, as the assumption of a perfectly adhering population is unrealistic in practice, we simulated alternative FIT screening strategies with longer intervals between test rounds, which are equivalent to reduced uptake of 60%, 40%, and 20% of offered FIT screening: (1) 3 FITs at ages 50, 52, and 54 (corresponding to 3 out of 5 tests), (2) 2 FITs at ages 50 and 53 (2 out of 5), and (3) 1 FIT at age 50 (1 out of 5), all of these followed by screening colonoscopy at age 55. These scenarios were considered appropriate to cover the range of adherence rates to offered FIT screening observed in practice, which range from approximately 20% in Germany to more than 60% in the Netherlands [15–17].

In all scenarios, individuals only received the next FIT test round if the respective previous test round was negative (e.g., in the model of current FIT screening offered in Germany, individuals only received a FIT at age 51 if the FIT at age 50 was negative, only received a FIT at age 52 if the FIT at age 51 was negative, and so forth), and individuals were only eligible for screening colonoscopy at age 55 if all previous FITs were negative (assuming that positive FITs had been directly followed by diagnostic colonoscopy).

#### Outcomes

First, we compared the model outcomes in terms of prevalences of any advanced neoplasms (advanced adenomas and cancers) and of cancers detected at a screening colonoscopy at age 55 for individuals for whom all FITs were negative, and we calculated the associated NNS. To assess the trajectories of possible colonoscopy detection rate and NNS, we also estimated models up to the age of 64 years in which the age at first screening colonoscopy was varied between 55 and 64 years.

Second, we assessed the PPV (the likelihood that an individual with a positive test truly has any advanced neoplasm or cancer) and the NPV (the likelihood that an individual with a negative test truly does not have any advanced neoplasm or cancer) for each of the 5 rounds of annual FIT testing. In addition, we also calculated the detection rate and NNS of the diagnostic colonoscopy following a positive FIT for each test round.

We defined our primary analysis of the German FIT–colonoscopy sequence assuming a perfectly adhering population. Findings based on this assumption are most valuable from the perspective of an individual seeking to minimize CRC risk by making use of the maximal screening offered. An assessment assuming full adherence is also relevant from a public health point of view, as an evaluation of conceptual strategies assuming imperfect adherence could lead to giving preference to strategies with short intervals between screens, to compensate for low population-level adherence, and potentially lead to over-screening in individuals perfectly adhering to offered screening, possibly resulting in unnecessary costs, risks, and test burden [18].

### Sensitivity analyses

We conducted 3 sets of one-way sensitivity analyses to explore the impact of uncertainty related to key parameters used in the model. First, all point estimates of the starting prevalences and transition rates were replaced by either the lower or upper limits of the 95% confidence intervals (CIs). Second, FIT sensitivities and specificities were assumed to be an absolute 5 percentage points lower or higher than in the base case scenario. Third, in order to account for potential dependencies between repeated rounds of FIT testing, we divided the study population in 2 groups, a group of 20% who were assumed to never have a positive FIT result and a group of 80% for whom FIT testing was assumed to have a 25% higher sensitivity and a 25% higher false positive rate than in the base case model. These assumptions assume the overall sensitivity and specificity to be unchanged, while allowing for differences in proneness to bleed across screening participants.

We considered these sets of one-way sensitivity analyses to be best fit for purpose for our study (as compared to a complex multi-way sensitivity analysis), as they allowed us to more specifically address the need of decision makers to understand the impact that changing the value of 1 specific parameter has on the results of the analysis.

The statistical software R (version 3.6.3) was used to set up the model and for all analyses. The R code defining the core model and additional scripts used for this study are in S1 Appendix.

### Patient and public involvement

Patients and the public were involved neither in the design and conduct of this modeling study, nor in the writing or editing of this document. Research at the German Cancer Research Center (DKFZ) is generally informed by a Patient Advisory Committee.

## Results

### Detection rates and NNS at age 55

Table 2 shows the expected detection rate of advanced neoplasm at screening colonoscopy at age 55 and the associated NNS after annual FIT testing at ages 50–54 compared to no preceding FIT testing and alternative FIT screening strategies. Of the simulated population of 100,000 men and 100,000 women with preceding annual FIT screening, 52% of men and 74% of women were eligible for screening colonoscopy at age 55. In these, the colonoscopy detection rate (associated NNS) at age 55 was 3.7% (27) and 0.10% (1,021) for any advanced neoplasm and cancer, respectively, in men, and 2.1% (47) and 0.05% (1,880) for any advanced neoplasm and cancer, respectively, in women.

**Table 2:** Expected detection rate and associated NNS to detect 1 case of any advanced neoplasm and cancer at screening colonoscopy at age 55 after annual FIT testing from age 50 to age 54, compared to no preceding FIT testing and to strategies with longer intervals between FITs.

Group	Proportion eligible for screening colonoscopy at age 55^1^	Any advanced neoplasm		Colorectal cancer	
		Detection rate	NNS	Detection rate	NNS
**Men**	** **	** **	** **	** **	** **
No preceding FIT screening	97%	9.7%	10	0.82%	122
Preceding FIT screening^2^					
Annually from age 50 to 54	52%	3.7%	27	0.10%	1,021
At ages 50, 52, and 54	66%	5.3%	19	0.17%	598
At ages 50 and 53	75%	6.6%	15	0.31%	322
At age 50	85%	8.2%	12	0.58%	173
**Women**					
No preceding FIT screening	98%	5.5%	18	0.40%	250
Preceding FIT screening^2^					
Annually from age 50 to 54	74%	2.1%	47	0.05%	1,880
At ages 50, 5,2 and 54	82%	3.0%	33	0.09%	1,104
At ages 50 and 53	87%	3.7%	27	0.16%	617
At age 50	93%	4.6%	22	0.29%	348

Simulated for a hypothetical cohort of 100,000 men and 100,000 women.

^1^Proportion of the initially simulated 100,000 individuals who are still alive, had no positive FIT, and no diagnosed CRC after all rounds of FIT testing.

^2^Assumptions: only individuals with negative FIT receive another FIT in the next round. Conditional independence between repeated rounds of FIT testing.

CRC, colorectal cancer; FIT, fecal immunochemical test; NNS, number needed to scope.

Detection rates and NNS changed markedly when FIT strategies with longer intervals between test rounds were used. With 3 preceding negative FITs at ages 50, 52, and 54, still approximately 600 and 1,100 colonoscopies were needed in men and women, respectively, to detect 1 case of cancer. In women, the NNS for detecting 1 cancer was still greater than 600 even with only 2 FITs at ages 50 and 53.

### Trajectory when postponing colonoscopy

**Fig 2:**
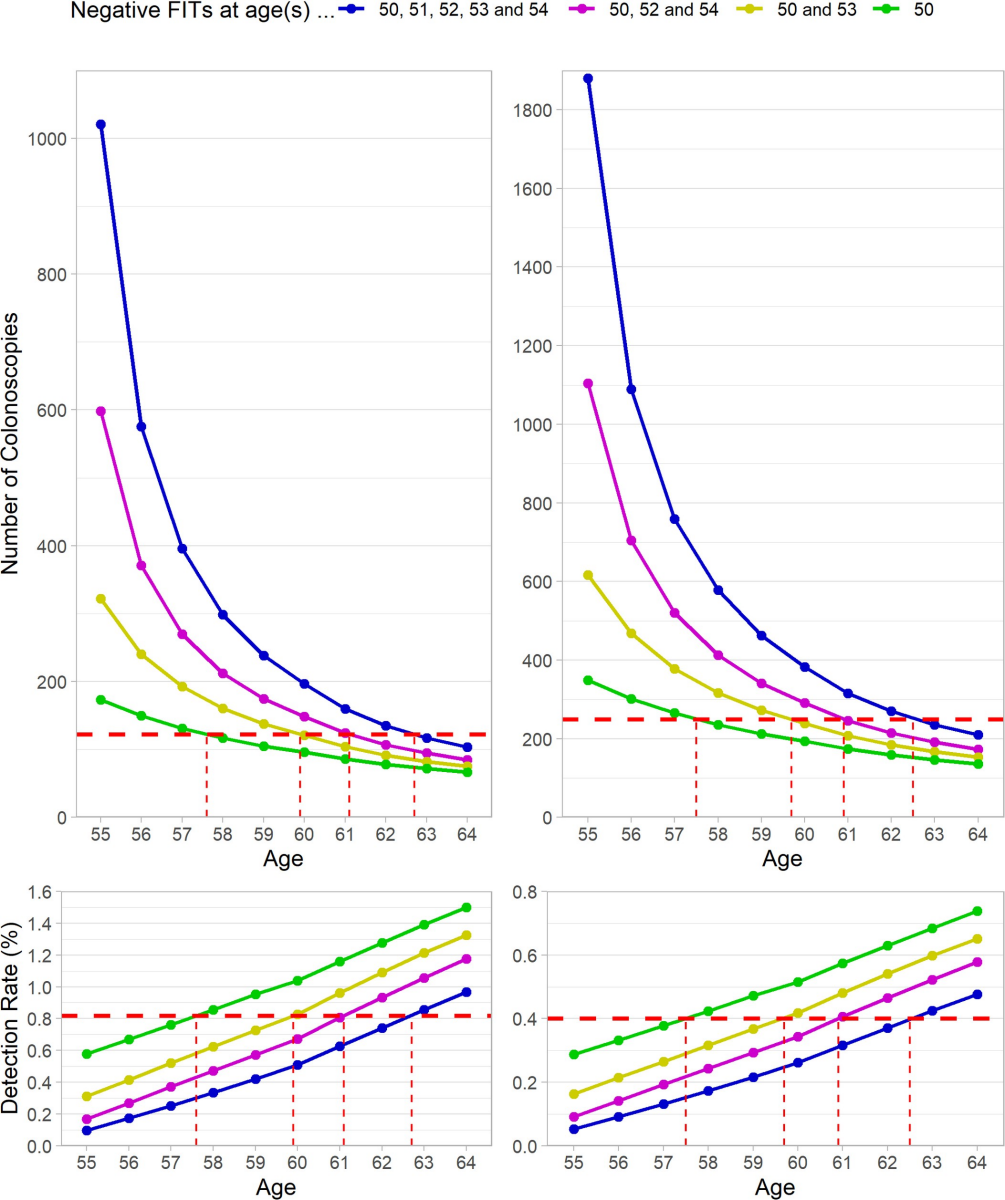
Trajectory of expected detection rate and associated NNS to detect 1 case of colorectal cancer with varying age at first screening colonoscopy. Top: NNS. Bottom: detection rate. Left: men. Right: women. Dashed horizontal red lines indicate the detection rate and NNS of previously unscreened individuals at a colonoscopy at age 55. Dashed vertical red lines indicate the age at which individuals with previously negative FIT screening reach the detection rate and NNS of those previously unscreened.FIT, fecal immunochemical test; NNS, number needed to scope.

Fig 2 shows the detection rates and associated NNS for CRC with varying age at first screening colonoscopy following preceding negative FIT screening, as compared to the detection rate and NNS at age 55 for a population without preceding FIT screening. Corresponding estimates for any advanced neoplasm can be seen in S1 Fig. Detection rates among those with 5 consecutive negative FITs were at a comparable level approximately 7–8 years later, i.e., at age 62–63, for both sexes.

### Performance of individual FIT test rounds

**Fig 3:**
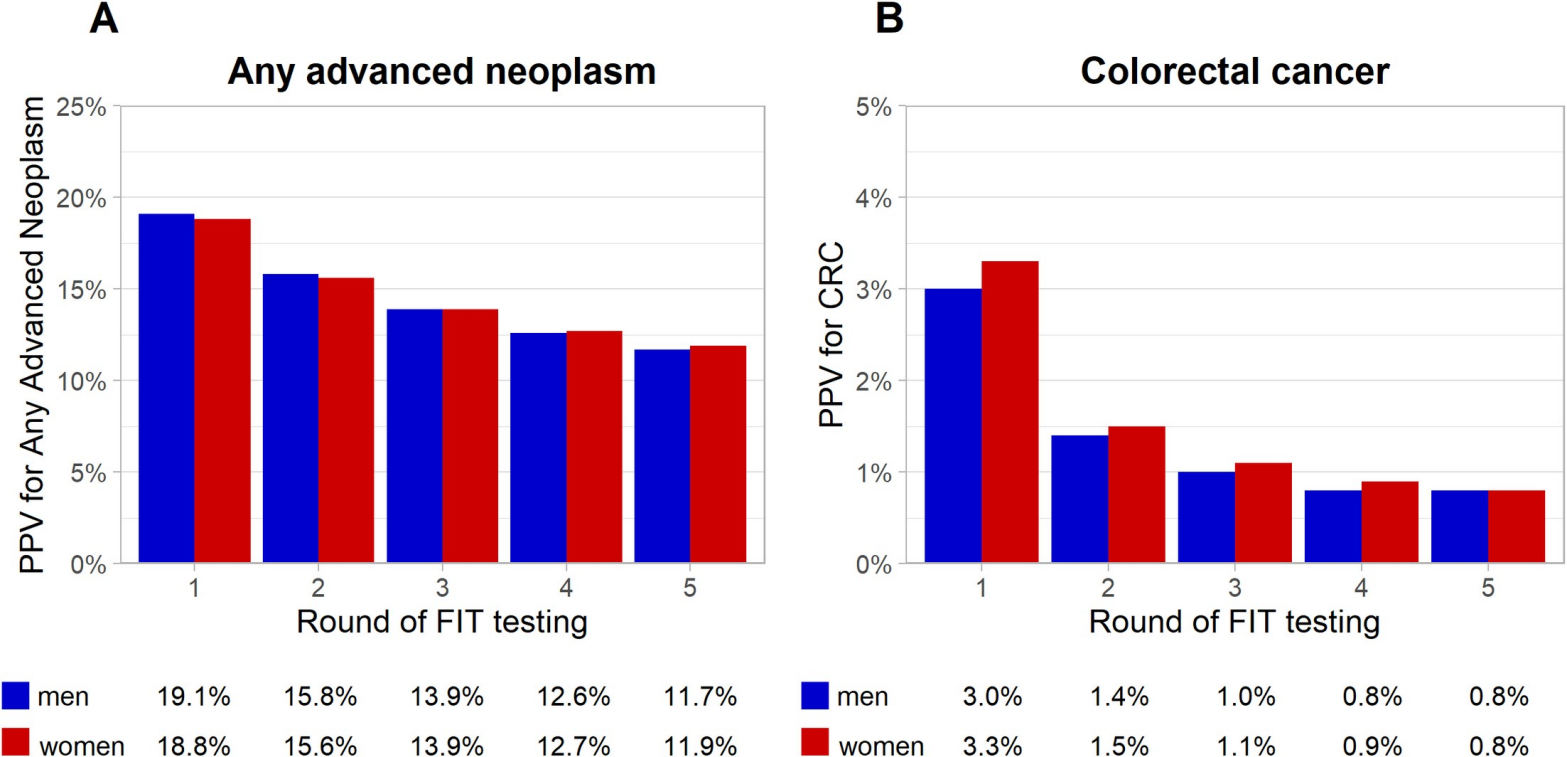
PPV for any advanced neoplasm and cancer over 5 rounds of annual FIT testing at ages 50–54 years, stratified by sex. (A) Any advanced neoplasm. (B) cancer. CRC, colorectal cancer; FIT, fecal immunochemical test; PPV, positive predictive value.

S4 Table gives an overview of the PPVs and NPVs for any advanced neoplasms and cancers over 5 rounds of annual FITs, stratified by sex and test round. It also shows the colonoscopy detection rates and associated NNS of diagnostic colonoscopies in individuals with a positive FIT by test round. PPV for any advanced neoplasm decreased from 19.1% and 18.8% to 11.7% and 11.9% for men and women, respectively, and PPV for cancer decreased from 3.0% and 3.3% to 0.8% and 0.8% for men and women, respectively (Fig 3). The NNS for diagnostic colonoscopy to detect 1 case of cancer ranged from 35 to 140 in men and from 31 to 130 in women.

### Sensitivity analyses

Results of sensitivity analyses are shown in S5 Table, S2 Fig, and S3 Fig. In sensitivity analyses using lower and upper limits of 95% CIs of starting prevalences and annual transition rates, the NNS for detecting 1 case of cancer at colonoscopy at age 55 after 5 negative FITs ranged from 818 to 1,312 in men and from 1,449 to 2,499 in women. Results appeared less robust when assuming 5-percentage-point lower or higher FIT diagnostic performance parameters (NNS: 627–1,774 in men, 1,120–3,118 in women) and when accounting for potential dependencies between repeated rounds of FIT testing by assuming differences in proneness to bleed across screening participants (NNS: 308 in men, 777 in women).

## Discussion

This study provided estimates of the diagnostic performance of the sequence of annual FIT screening at ages 50–54 followed by colonoscopy screening at age 55, as currently offered in Germany. The sequential approach may lead to an inappropriate use of screening, as perfect adherence is associated with rapidly decreasing PPV of consecutive FITs and promotes subsequent screening colonoscopy in individuals who are very unlikely to benefit. In particular, PPV for cancer was decreased to approximately 1% for both sexes already after the third round of testing, having dropped by approximately two-thirds from the first round. After 5 consecutive negative FITs, colonoscopy detection rates for cancer at age 55 were ≤0.10% for both sexes, with associated NNS of approximately 1,900 for women and of >1,000 for men, approximately 8 times the NNS for a previously unscreened population. Estimates for NNS were markedly reduced in alternative strategies involving longer FIT screening intervals.

### Findings in context

Considering the increasing demand for colonoscopy resources due to the implementation of nationwide organized screening for CRC, along with the demographic aging process in Germany, strategic use of colonoscopy should be a priority. Albeit rarely, colonoscopy can cause complications [19,20] and requires major resources. Screening in individuals unlikely to benefit comes at the cost of both, inducing unnecessary test burden, harms, expenses for the healthcare system, and demand of colonoscopy capacities.

There are 3 points of concern regarding the design of the screening offered in Germany. First, the evidence base for offering intensive annual FIT testing scheme only in the age group 50–54 years is unclear. The vast majority of randomized controlled trial evidence on fecal testing assessed a biennial interval [3]. These studies assessed the guaiac-based fecal occult blood test (gFOBT), while the diagnostic performance of FIT was shown to be considerably better than that of gFOBT [21–23]. In addition, results from a Dutch randomized study comparing different FIT intervals raised doubts regarding an additional benefit of using an annual instead of a biennial scheme [24]. More recent evidence suggests that intervals might even be safely extended to up to 5 years [25]. Longer intervals are also supported by the findings of our study, as, even with 2 preceding FITs at ages 50 and 53, still approximately 300 colonoscopies in men at age 55, and 600 colonoscopies in women, would be required to detect 1 case of cancer. Finally, considering that the risk for CRC increases strongly with older age [11], it seems contradictory to offer the individuals aged 50–54 years more intensive screening than individuals in older age groups.

Second, the logic of the age-dependent differences in offered screening seems questionable. This is particularly true for women, who are only offered FIT screening at ages 50–54 and who have been shown to have higher screening uptake than men [26]. Even though full adherence, as assumed in our study, is unrealistic, potential use may be substantial, as >70% of women in our simulations had 5 preceding negative FITs before they were eligible to receive screening colonoscopy at age 55. In addition, women have lower risk of developing adenomas and CRC than men [27]. In our simulation, this resulted in NNS estimates approximately twice as high in women than in men across all simulated scenarios. These sex-related differences should be given careful consideration when designing screening strategies. For instance, intensive screening may be more beneficial for men, but involves a high potential for over-screening in women, which suggests large potential for sex-differentiated approaches.

Third, as the sensitivity of FIT tests is high for preclinical cancers but limited for CRC precursor lesions, FITs are useful to lower the mortality burden of late-stage cancers, but less so to reduce the CRC incidence rate. Screening colonoscopy, on the other hand, is the gold standard to lower CRC mortality and incidence (both stated objectives of the German screening program [28]) as it allows detection and removal of precursor lesions at high sensitivity directly upon examination. Reaching the objective of reducing the CRC incidence rate by focusing first on FIT testing may therefore be associated with an unnecessary high burden of testing, as either intensive, but rather inefficient, FIT testing or eventually performing screening colonoscopy is required.

Our findings suggest that a prevalence of advanced neoplasm comparable to that of a previously unscreened population would be reached 7–8 years after the last negative FIT if 5 screening tests are used, and some years earlier with less intensive FIT screening. Interestingly, even with only 1 FIT at age 50, the advanced neoplasm prevalence of a previously unscreened population was only reached at age 57.

This indicates that longer intervals between FITs and colonoscopy may potentially be more appropriate than colonoscopy at age 55 following negative preceding FITs. However, it remains unclear whether delaying the 2 screening colonoscopies 10 years apart that are offered in the German screening program would be associated with an increase of prevented CRC deaths in the long run, regardless of the previous FIT screening strategy, and compared to conventional screening strategies based on only 1 screening modality (e.g., only colonoscopy). Furthermore, FIT-based screening at ages 50–54 shifts the 2 offered colonoscopies from ages 50 and 60 to ages 55 and 65, which was previously estimated to be associated with preventing fewer CRC deaths [14]. To what extent this disadvantage can be compensated by the additional FITs is unknown. While clearly of interest, modeling comparative long-term effectiveness was beyond the scope of this study, which had the objective of assessing a currently offered FIT–colonoscopy screening strategy in terms of key parameters of diagnostic performance. Further research should investigate the long-term performance of FIT–colonoscopy sequencing approaches as compared to conventional strategies relying on only 1 screening modality.

Finally, our primary analysis of the performance of the German FIT–colonoscopy sequence relies on the hypothetical situation of complete adherence, in particular for the 5 consecutive annual FITs. Although high adherence rates to FIT-based screening have been achieved in countries with well-organized screening programs, such as the Netherlands [15,16], participation rates have remained low in Germany. For instance, estimates for the use of at least 1 fecal stool test in the period 2015–2016 were only at 21% and 18% for all eligible women and men, respectively [17], which implies that the scenario of perfect adherence modeled in this study does not reflect current practice. Given the low overall FIT adherence rate in Germany, the proportion undergoing screening colonoscopy after 5 consecutive negative FITs is likely small. Although this could be interpreted as being reassuring in the light of the results of our study, such “assurance” would be based on mutual compensation of major deficits in the design of screening schemes that would lead to inefficient use of screening resources in cases of high adherence on the one hand, and major deficits in adherence to those schemes on the other hand. A much more rational alternative would be to offer meaningful screening schemes in an organized manner that ensures high adherence to such schemes.

### Strengths and limitations

A major strength of this study is the use of model parameters derived from data specifically collected from the German population, including data from a large screening colonoscopy registry. Major limitations concern simplifying model assumptions and uncertainties related to input parameters. For instance, as the true adenoma miss rate at colonoscopy in Germany is unknown, we used representative estimates derived from a comprehensive systematic review and meta-analysis using data not limited by geographic region [29]. Uncertainties also relate to the true diagnostic performance of FIT testing and potential differences between sexes in this respect, which we however sought to address by providing comprehensive sensitivity analyses. Finally, due to the lack of relevant data, the proportions of neoplasms and transition rates between states among people aged 50 years were assumed to be the same as those among people aged 55. However, potential bias from violation of this assumption would likely be small, given that very similar prevalences of neoplasms were observed in age groups 50–54 and 55–59 in regional programs offering screening colonoscopy from age 50 on [30], and variation of transition rates by age was generally small.

### Conclusion

Screening colonoscopy at age 55 after 5 consecutive negative FITs at age 50–54, as currently offered in the German cancer early detection program, is expected to have very low PPV. Our results may inform efforts to enhance the design of screening programs. Further research should focus on the comparative long-term effectiveness of current screening approaches in Germany and assess potentially more rational approaches of sequencing CRC screening tests.

## Supporting information

S1 Appendix Model source code. (ZIP)

S1 Fig Trajectory of expected detection rate and associated NNS to detect 1 case of any advanced neoplasm with varying age at screening colonoscopy. (DOCX)

S2 Fig Sensitivity analysis: Trajectory of expected detection rate and associated NNS to detect 1 case of any advanced neoplasm or 1 case of cancer with varying age at screening colonoscopy. (DOCX)

S3 Fig Sensitivity Analysis: PPV for any advanced neoplasm and cancer over 5 rounds of annual FIT testing at ages 50–54. (DOCX)

S1 Table Overview of model parameters. (DOCX)

S2 Table Annual CRC-specific mortality rates of CRC patients by mode of cancer detection. (DOCX)

S3 Table Sex- and age-specific general mortality rates. (DOCX)

S4 Table PPV and NPV of 5 consecutive annual FITs at ages 50–54, and numbers of diagnostic colonoscopies after a positive FIT needed to detect 1 case of any advanced neoplasm or 1 CRC, stratified by sex and test round. (DOCX)

S5 Table Sensitivity Analysis: Expected detection rate and associated NNS to detect 1 case of any advanced neoplasm or cancer at screening colonoscopy at age 55 after annual FIT testing from age 50 to age 54, compared to no preceding FIT testing and to strategies with longer intervals between FITs. (DOCX)

S1 Text Model documentation. (DOCX)

## Abbreviations

CRC: colorectal cancer
FIT: fecal immunochemical test
NNS: number needed to scope
NPV: negative predictive value
PPV: positive predictive value

## References

[1] Bray F, Ferlay J, Soerjomataram I, Siegel RL, Torre LA, Jemal A. Global cancer statistics 2018: GLOBOCAN estimates of incidence and mortality worldwide for 36 cancers in 185 countries. CA Cancer J Clin. 2018;68:394–424. 10.3322/caac.21492 30207593

[2] Zentrum für Krebsregisterdaten. Datenbankabfrage. Berlin: Zentrum für Krebsregisterdaten; 2020 [cited 2020 Jun 29]. Available from: https://www.krebsdaten.de/Krebs/DE/Datenbankabfrage/datenbankabfrage_stufe1_node.html.

[3] Hewitson P, Glasziou P, Watson E, Towler B, Irwig L. Cochrane systematic review of colorectal cancer screening using the fecal occult blood test (hemoccult): an update. Am J Gastroenterol. 2008;103:1541–9. 10.1111/j.1572-0241.2008.01875.x 18479499

[4] Heitman SJ, Hilsden RJ, Au F, Dowden S, Manns BJ. Colorectal cancer screening for average-risk North Americans: an economic evaluation. PLoS Med. 2010;7:e1000370 10.1371/journal.pmed.1000370 21124887 PMC2990704

[5] Lansdorp-Vogelaar I, Knudsen AB, Brenner H. Cost-effectiveness of colorectal cancer screening—an overview. Best Pract Res Clin Gastroenterol. 2010;24:439–49. 10.1016/j.bpg.2010.04.004 20833348 PMC2939039

[6] Tao S, Hoffmeister M, Brenner H. development and validation of a scoring system to identify individuals at high risk for advanced colorectal neoplasms who should undergo colonoscopy screening. Clin Gastroenterol Hepatol. 2014;12:478–85. 10.1016/j.cgh.2013.08.042 24022090

[7] Senore C, Hassan C, Regge D, Pagano E, Iussich G, Correale L, et al Cost-effectiveness of colorectal cancer screening programmes using sigmoidoscopy and immunochemical faecal occult blood test. J Med Screen. 2019;26:76–83. 10.1177/0969141318789710 30180780

[8] Bénard F, Barkun AN, Martel M, von Renteln D. Systematic review of colorectal cancer screening guidelines for average-risk adults: summarizing the current global recommendations. World J Gastroenterol. 2018;24:124–38. 10.3748/wjg.v24.i1.124 29358889 PMC5757117

[9] Schreuders EH, Ruco A, Rabeneck L, Schoen RE, Sung JJ, Young GP, et al Colorectal cancer screening: a global overview of existing programmes. Gut. 2015;64:1637–49. 10.1136/gutjnl-2014-309086 26041752

[10] Brenner H, Altenhofen L, Stock C, Hoffmeister M. Prevention, early detection, and overdiagnosis of colorectal cancer within 10 years of screening colonoscopy in Germany. Clin Gastroenterol Hepatol. 2015;13:717–23. 10.1016/j.cgh.2014.08.036 25218160

[11] Brenner H, Altenhofen L, Stock C, Hoffmeister M. Expected long-term impact of the German screening colonoscopy programme on colorectal cancer prevention: analyses based on 4,407,971 screening colonoscopies. Eur J Cancer. 2015;51:1346–53. 10.1016/j.ejca.2015.03.020 25908273

[12] Brenner H, Kretschmann J, Stock C, Hoffmeister M. Expected long-term impact of screening endoscopy on colorectal cancer incidence: a modelling study. Oncotarget. 2016;7:48168–79. 10.18632/oncotarget.10178 27340865 PMC5217009

[13] Chen C, Stock C, Hoffmeister M, Brenner H. How long does it take until the effects of endoscopic screening on colorectal cancer mortality are fully disclosed?: a Markov model study. Int J Cancer. 2018;143:2718–24. 10.1002/ijc.31716 29978478

[14] Chen C, Stock C, Hoffmeister M, Brenner H. Optimal age for screening colonoscopy: a modeling study. Gastrointest Endosc. 2019;89:1017–25.e12. 10.1016/j.gie.2018.12.021 30639539

[15] van Roon AHC, Hol L, Wilschut JA, Reijerink JCIY, van Vuuren AJ, van Ballegooijen M, et al Advance notification letters increase adherence in colorectal cancer screening: a population-based randomized trial. Prev Med. 2011;52:448–51. 10.1016/j.ypmed.2011.01.032 21457725

[16] Toes-Zoutendijk E, van Leerdam ME, Dekker E, van Hees F, Penning C, Nagtegaal I, et al Real-time monitoring of results during first year of Dutch colorectal cancer screening program and optimization by altering fecal immunochemical test cut-off levels. Gastroenterology. 2017;152:767–75.e2. 10.1053/j.gastro.2016.11.022 27890769

[17] Starker A, Buttmann-Schweiger N, Krause L, Barnes B, Kraywinkel K, Holmberg C. [Cancer screening in Germany: availability and participation.] 2018;61:1491–9. 10.1007/s00103-018-2842-8 30406892

[18] Knudsen AB, Zauber AG, Rutter CM, Naber SK, Doria-Rose VP, Pabiniak C, et al Estimation of benefits, burden, and harms of colorectal cancer screening strategies: modeling study for the US Preventive Services Task Force. JAMA. 2016;315:2595–609. 10.1001/jama.2016.6828 27305518 PMC5493310

[19] Rabeneck L, Paszat LF, Hilsden RJ, Saskin R, Leddin D, Grunfeld E, et al Bleeding and perforation after outpatient colonoscopy and their risk factors in usual clinical practice. Gastroenterology. 2008;135:1899–906. 10.1053/j.gastro.2008.08.058 18938166

[20] Stock C, Ihle P, Sieg A, Schubert I, Hoffmeister M, Brenner H. Adverse events requiring hospitalization within 30 days after outpatient screening and nonscreening colonoscopies. Gastrointest Endosc. 2013;77:419–29. 10.1016/j.gie.2012.10.028 23410698

[21] Faivre J, Dancourt V, Denis B, Dorval E, Piette C, Perrin P, et al Comparison between a guaiac and three immunochemical faecal occult blood tests in screening for colorectal cancer. Eur J Cancer. 2012;48:2969–76. 10.1016/j.ejca.2012.04.007 22572481

[22] Brenner H, Tao S. Superior diagnostic performance of faecal immunochemical tests for haemoglobin in a head-to-head comparison with guaiac based faecal occult blood test among 2235 participants of screening colonoscopy. Eur J Cancer. 2013;49:3049–54. 10.1016/j.ejca.2013.04.023 23706981

[23] Wieten E, Schreuders EH, Grobbee EJ, Nieboer D, Bramer WM, Lansdorp-Vogelaar I, et al Incidence of faecal occult blood test interval cancers in population-based colorectal cancer screening: a systematic review and meta-analysis. Gut. 2019;68:873–81. 10.1136/gutjnl-2017-315340 29934436

[24] van Roon AH, Goede SL, van Ballegooijen M, van Vuuren AJ, Looman CW, Biermann K, et al Random comparison of repeated faecal immunochemical testing at different intervals for population-based colorectal cancer screening. Gut. 2013;62:409–15. 10.1136/gutjnl-2011-301583 22387523

[25] Haug U, Grobbee EJ, Lansdorp-Vogelaar I, Spaander MCW, Kuipers EJ. Immunochemical faecal occult blood testing to screen for colorectal cancer: can the screening interval be extended? Gut. 2017;66:1262 10.1136/gutjnl-2015-310102 27006184

[26] Altenhofen L. Projekt Wissenschaftliche Begleitung von Früherkennungs-Koloskopien in Deutschland: Berichtszeitraum 2014. 12 Jahresbericht, Version 2. Berlin: Zentralinstitut für die kassenärztliche Versorgung in Deutschland; 2016 [cited 2020 Jun 30]. Available: https://www.zi.de/fileadmin/user_upload/Jahresbericht_2014_Darmkrebs_Frueherkennung.pdf.

[27] Brenner H, Altenhofen L, Stock C, Hoffmeister M. Incidence of colorectal adenomas: birth cohort analysis among 4.3 million participants of screening colonoscopy. Cancer Epidemiol Biomarkers Prev. 2014;23:1920–7. 10.1158/1055-9965.EPI-14-0367 25012996

[28] Federal Joint Committee. [Resolution of the Federal Joint Committee on the Directive for Organized Cancer Screening Programmes and an amendment of the Directive on Cancer Screening.] Berlin: Federal Joint Committee; 2018 [cited 2020 Jun 29]. Available from: https://www.g-ba.de/downloads/39-261-3418/2018-07-19_2018-08-02_oKFE-RL_Beschluss-oKFE-RL-Aenderung_KFE-RL_konsolidiert_BAnz.pdf

[29] Zhao S, Wang S, Pan P, Xia T, Chang X, Yang X, et al Magnitude, risk factors, and factors associated with adenoma miss rate of tandem colonoscopy: a systematic review and meta-analysis. Gastroenterology. 2019;156:1661–74.e11. 10.1053/j.gastro.2019.01.260 30738046

[30] Brenner H, Zwink N, Ludwig L, Hoffmeister M. Should screening colonoscopy be offered from age 50? Dtsch Arztebl Int. 2017;114:94–100. 10.3238/arztebl.2017.0094 28266302 PMC5341112

