# Supplementary material for: Age-specific sequence of colorectal cancer screening options in Germany: A model-based critical evaluation
Source: PLoS Med. 2020 Jul 17;17(7):e1003194. doi: 10.1371/journal.pmed.1003194 (PMC7367446; doi:10.1371/journal.pmed.1003194)
Supplement: S1 Fig — (DOCX) [file pmed.1003194.s002.docx]

#### **Supplementary Figure 1** Trajectory of expected detection rate and associated NNS to detect 1 case of any advanced neoplasm with varying age at first screening colonoscopy.


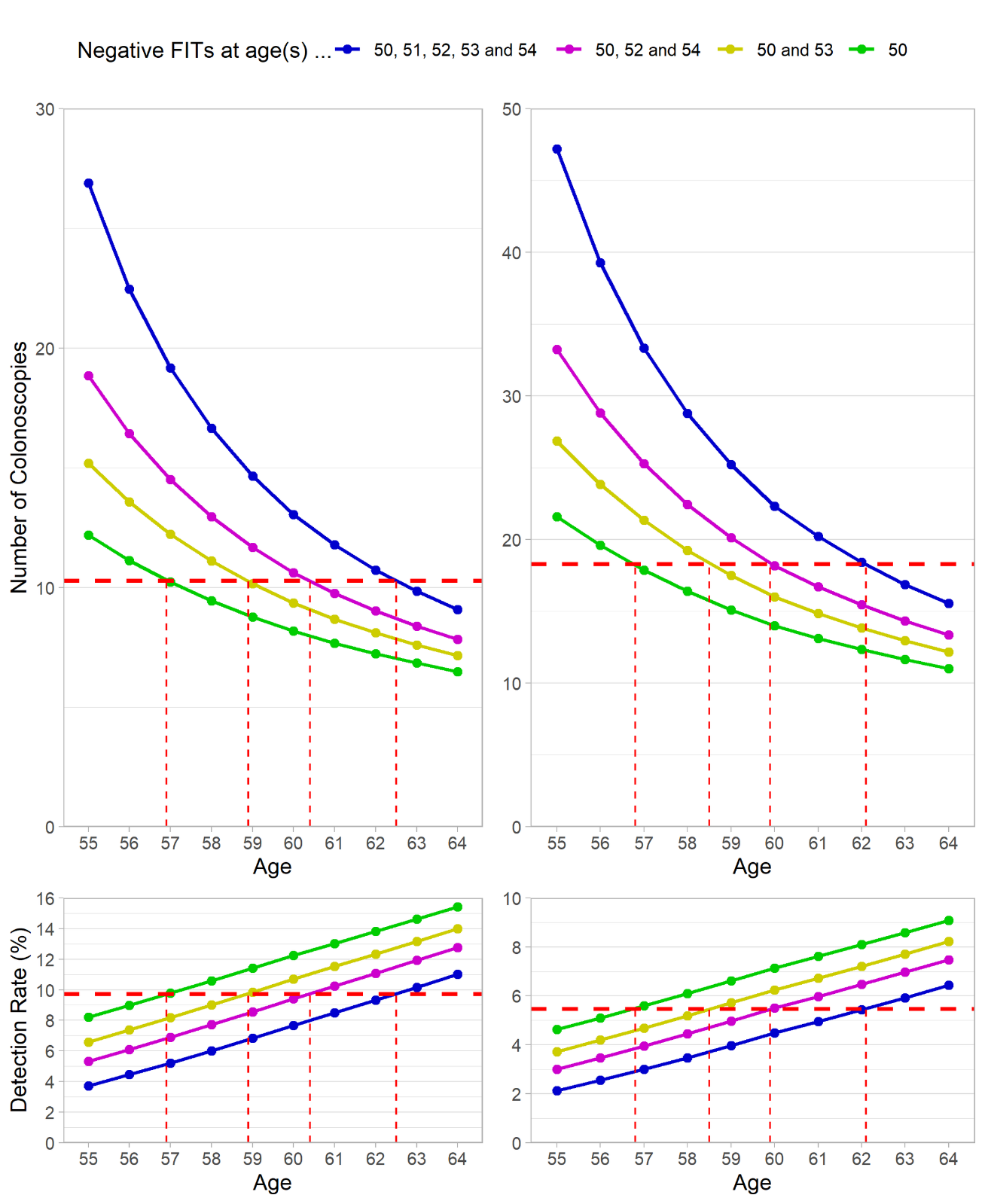


Top: NNS. Bottom: detection rate. Left: men. Right: women. Dashed horizontal red lines indicate the detection rate and NNS of previously unscreened individuals at a colonoscopy at age 55. Dashed vertical red lines indicate the age at which individuals with previously negative FIT screening reach the detection rate and NNS of those previously unscreened.

FIT, fecal immunochemical test; NNS, number needed to scope.
