# Supplementary material for: Age-specific sequence of colorectal cancer screening options in Germany: A model-based critical evaluation
Source: PLoS Med. 2020 Jul 17;17(7):e1003194. doi: 10.1371/journal.pmed.1003194 (PMC7367446; doi:10.1371/journal.pmed.1003194)
Supplement: S2 Fig — (DOCX) [file pmed.1003194.s003.docx]

#### **Supplementary Figure 2** Sensitivity analysis: Trajectory of expected detection rate and associated NNS to detect 1 case of any advanced neoplasm or 1 case of cancer with varying age at screening colonoscopy. Top: NNS. Bottom: detection rate. Left: men. Right: women.

##### **A1. Starting Prevalences and Transition Rates Lower Limit**

| **Any advanced neoplasm** | **Colorectal cancer** |
| --- | --- |
| 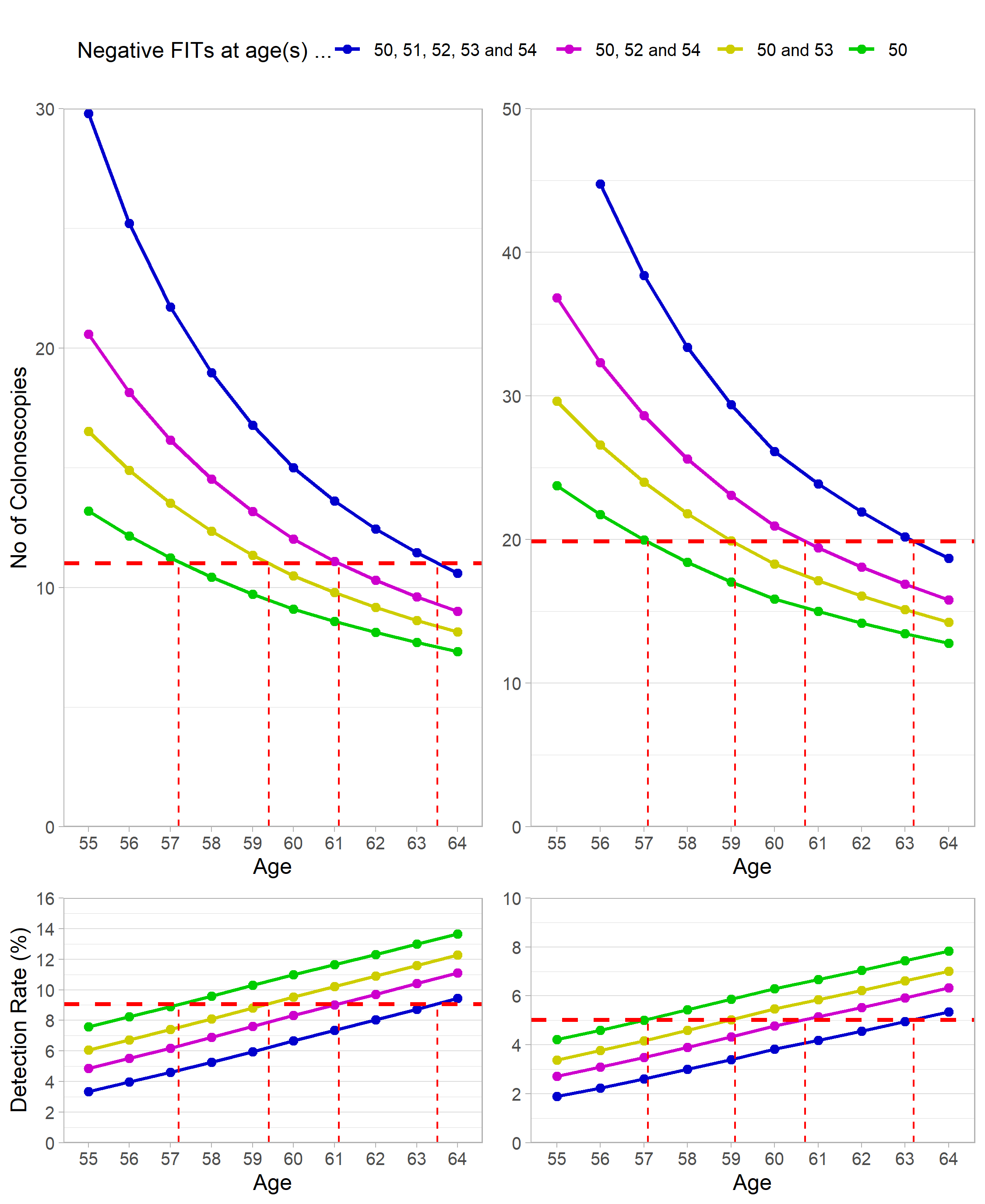 | 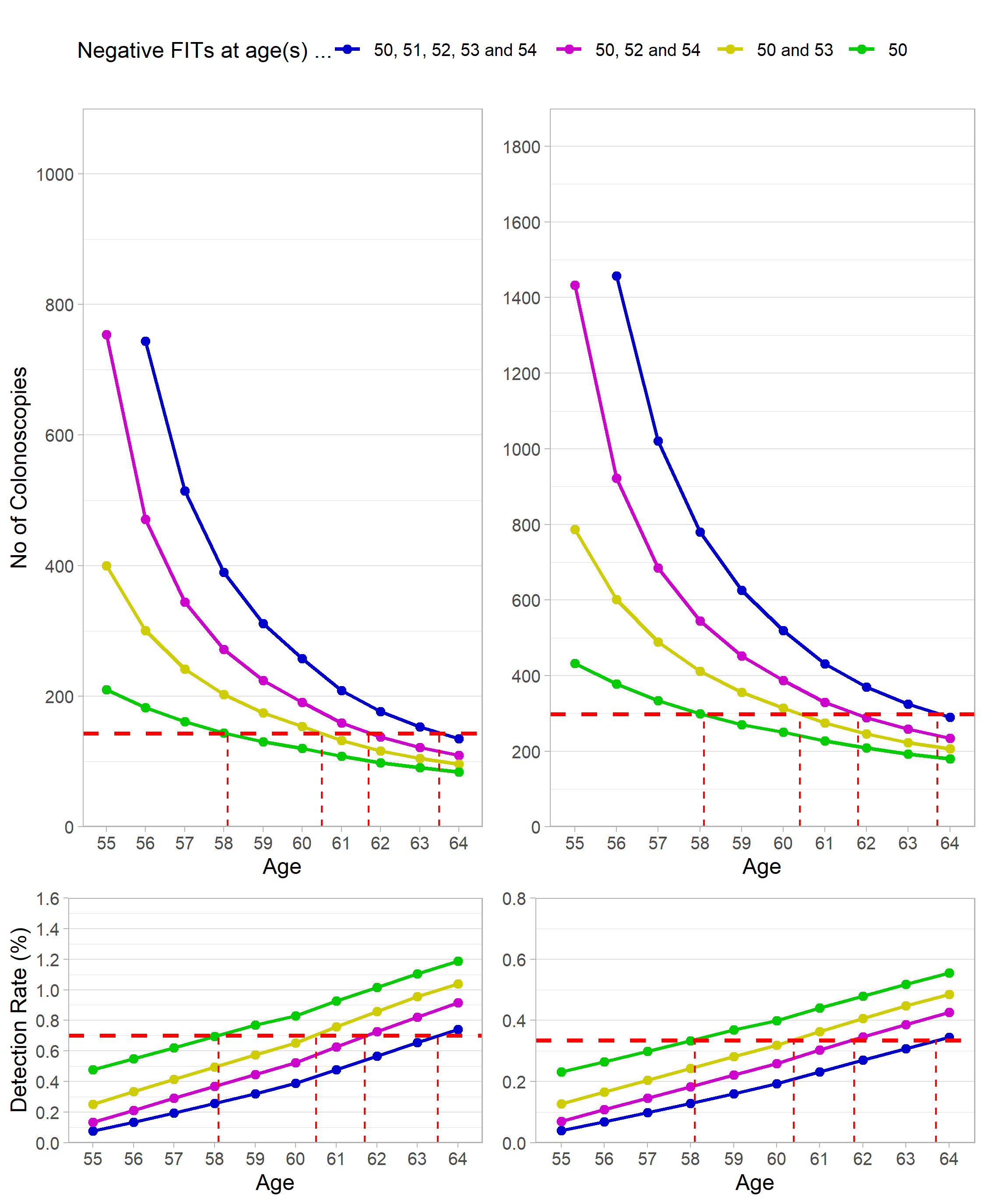 |

##### **A2. Starting Prevalences and Transition Rates Upper Limit**

| **Any advanced neoplasm** | **Colorectal cancer** |
| --- | --- |
| 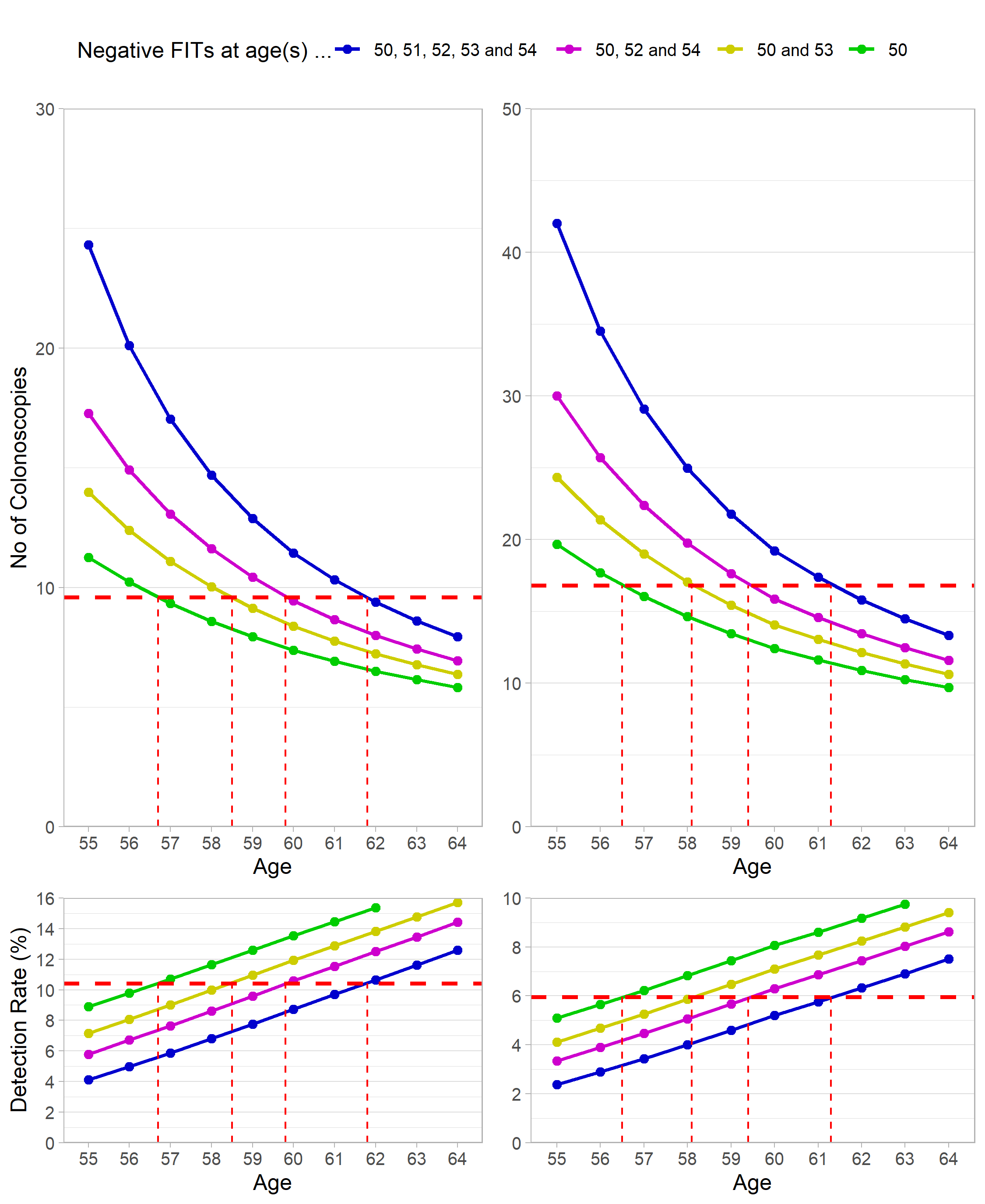 | 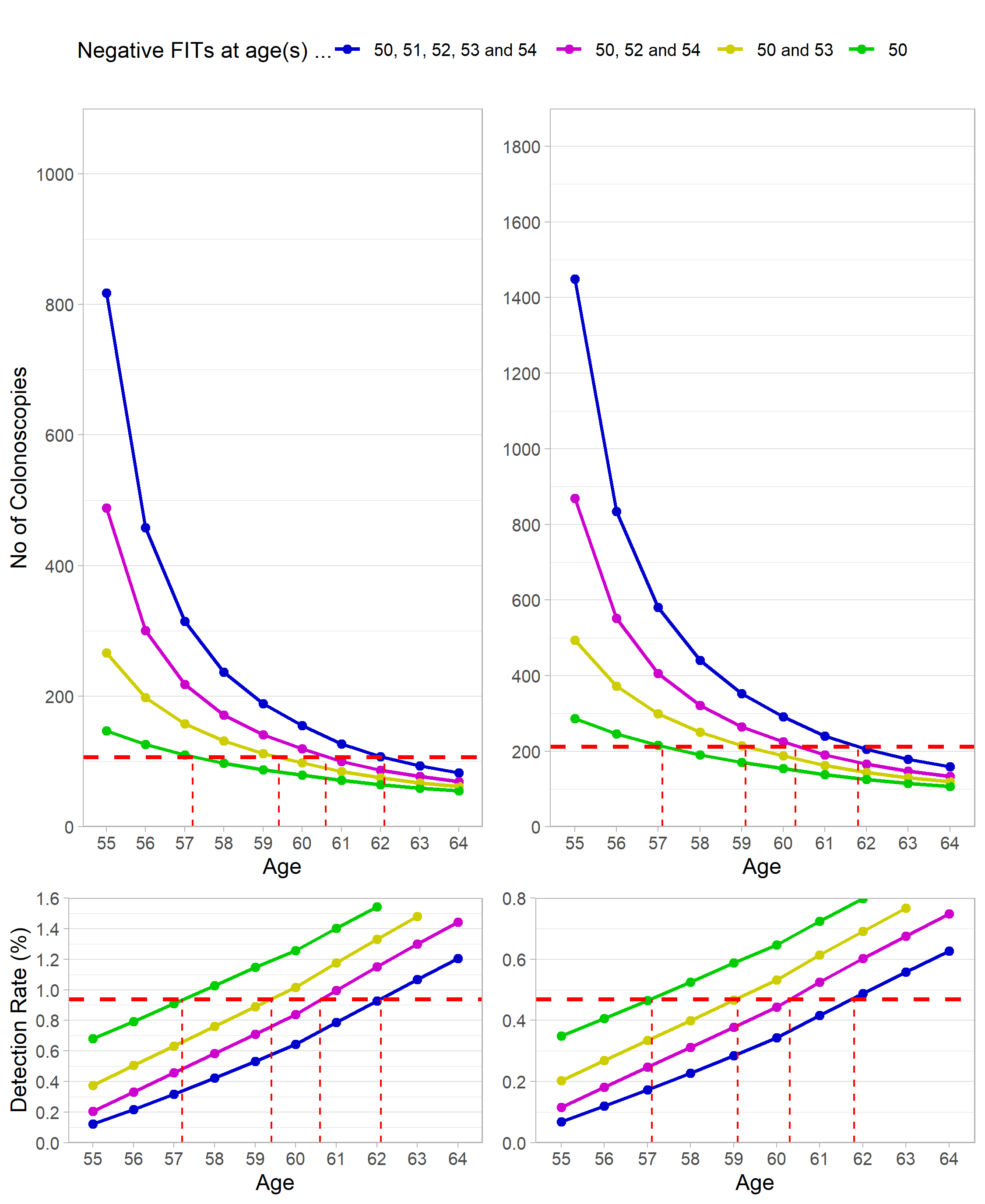 |

##### **B1. FIT Sensitivity and Specificity Absolute Five Per Cent Points Lower**

| **Any advanced neoplasm** | **Colorectal cancer** |
| --- | --- |
| 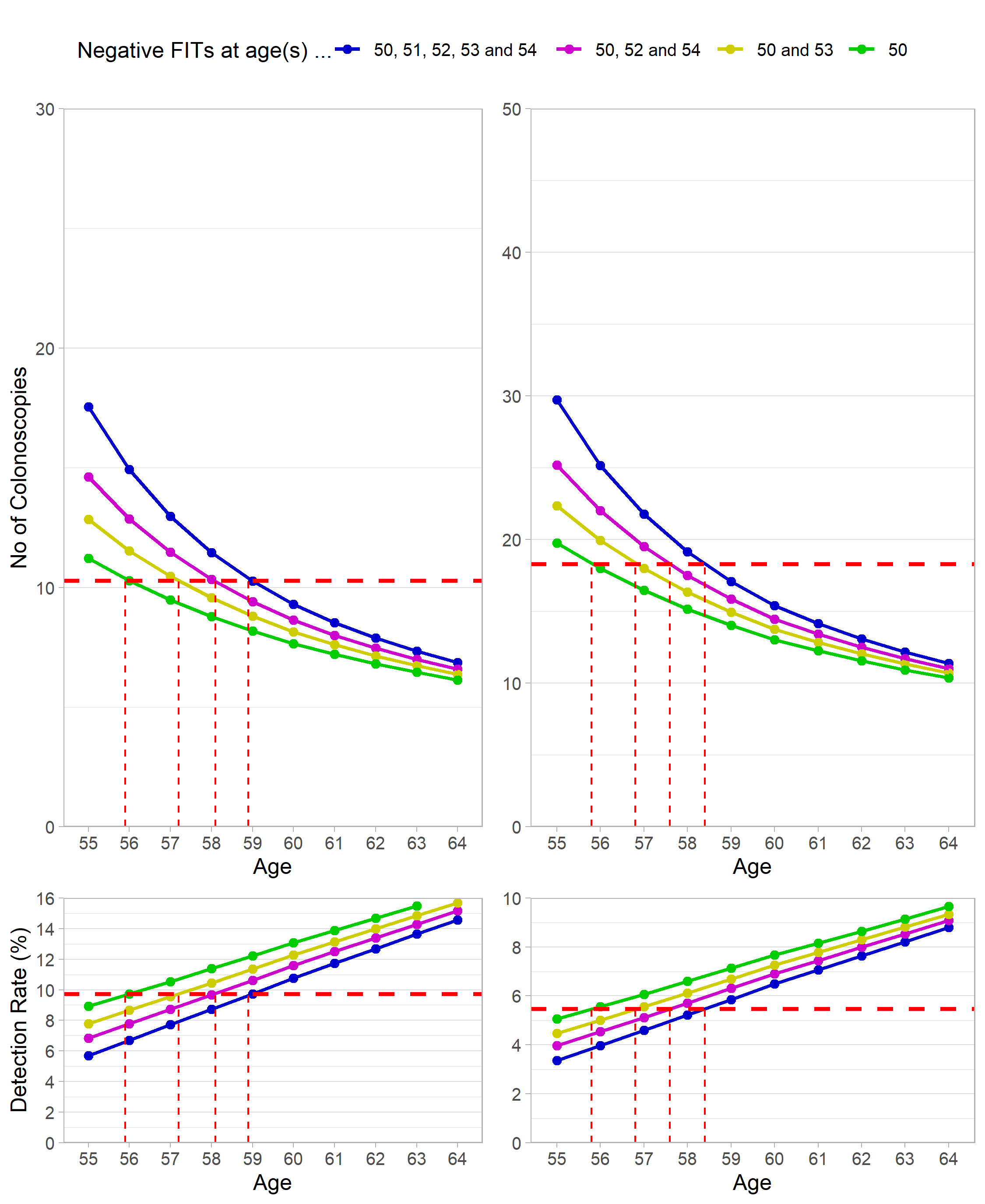 | 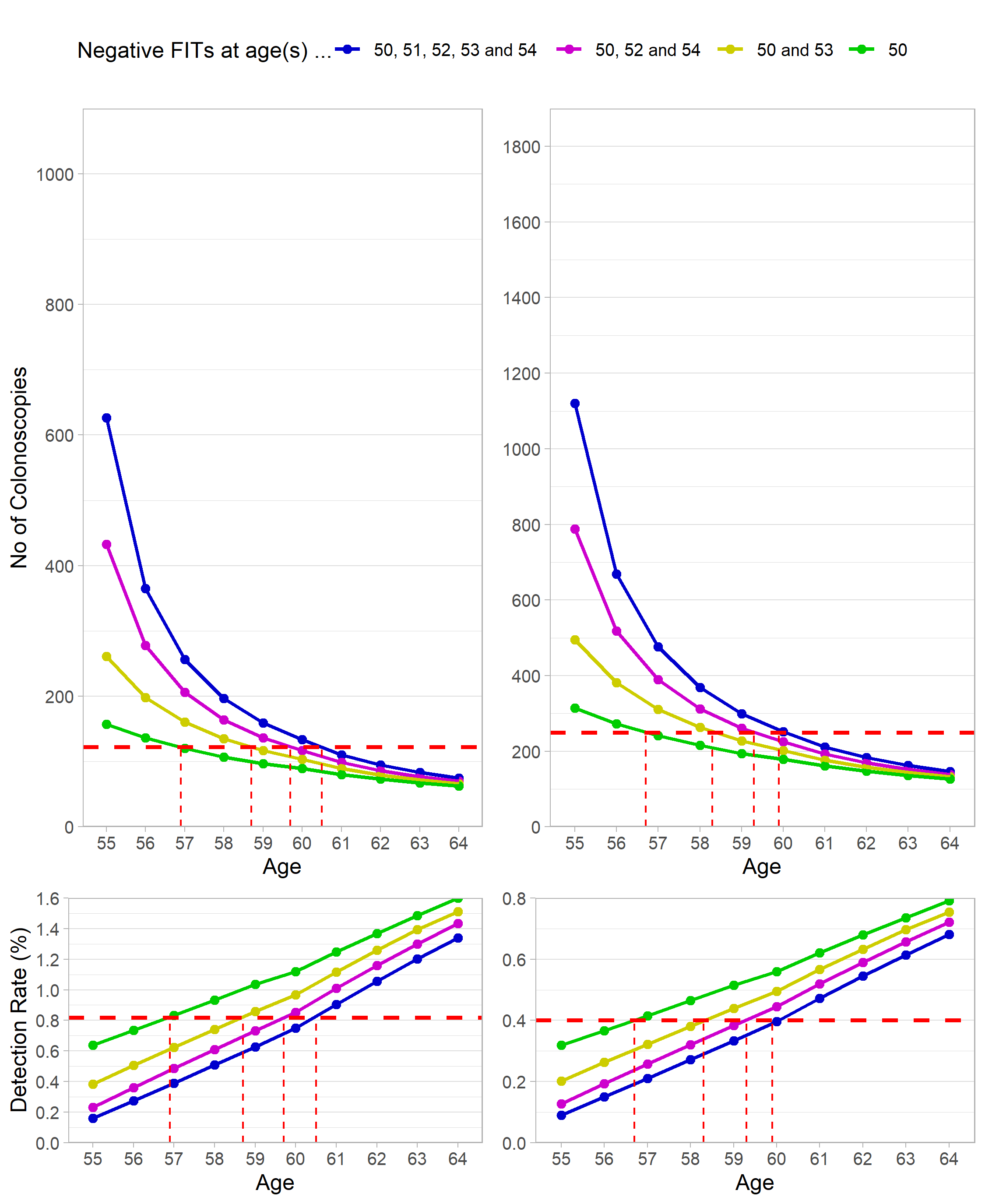 |

##### **B2. FIT Sensitivity and Specificity Absolute Five Per Cent Points Higher**

| **Any advanced neoplasm** | **Colorectal cancer** |
| --- | --- |
| 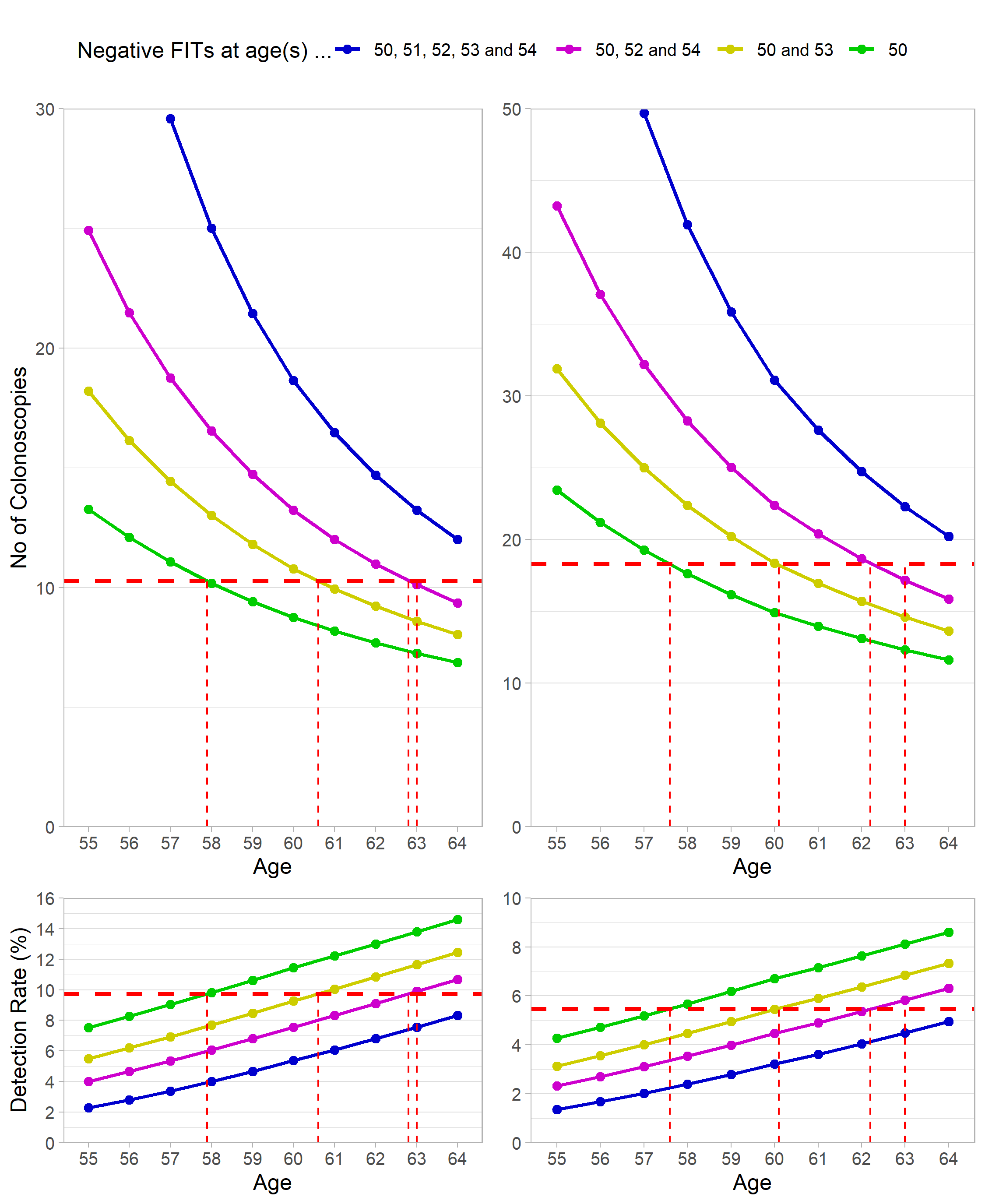 | 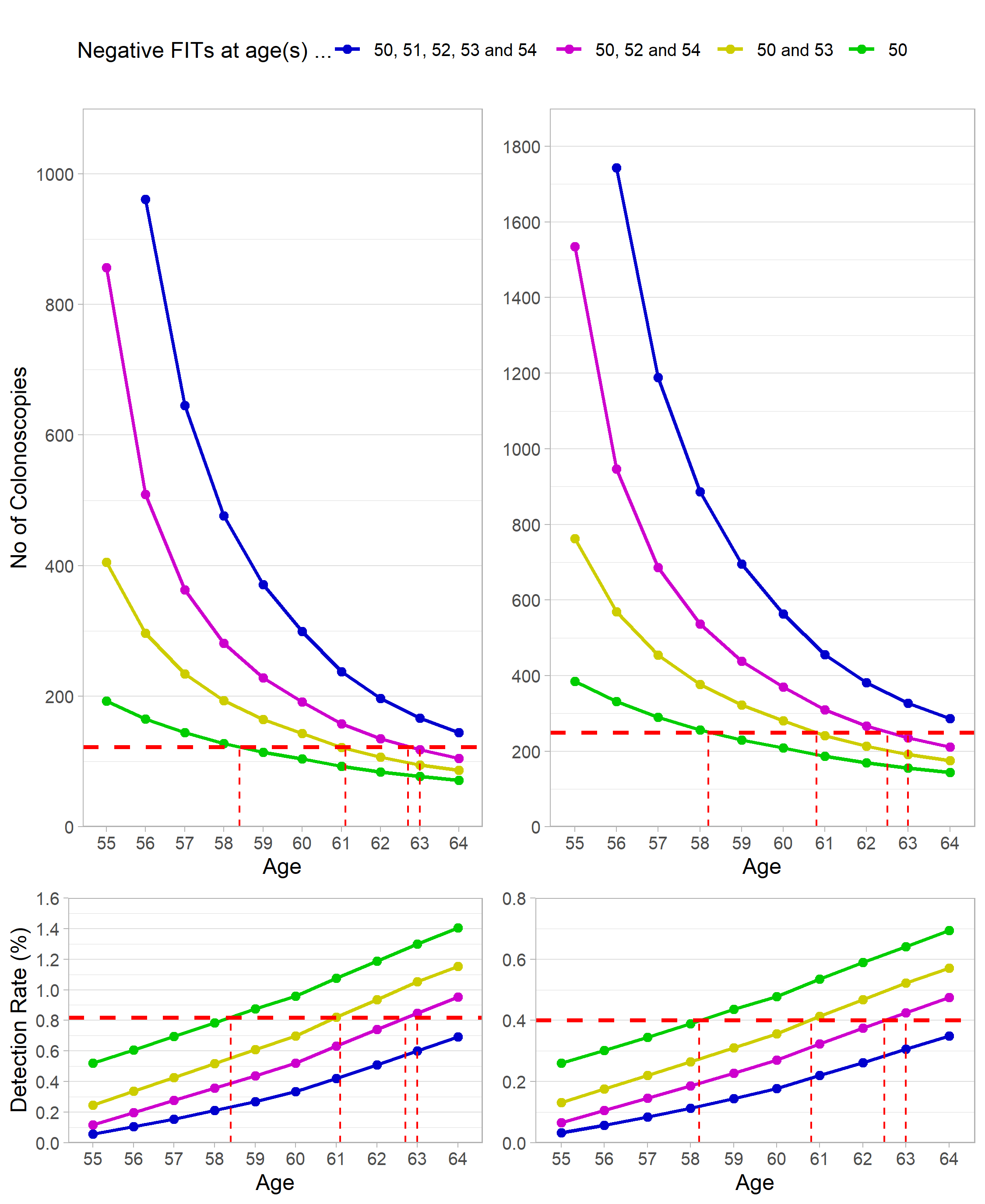 |

##### **C. Dependencies (Differences In Proneness To Bleed Across Screenees)**

| **Any advanced neoplasm** | **Colorectal cancer** |
| --- | --- |
| 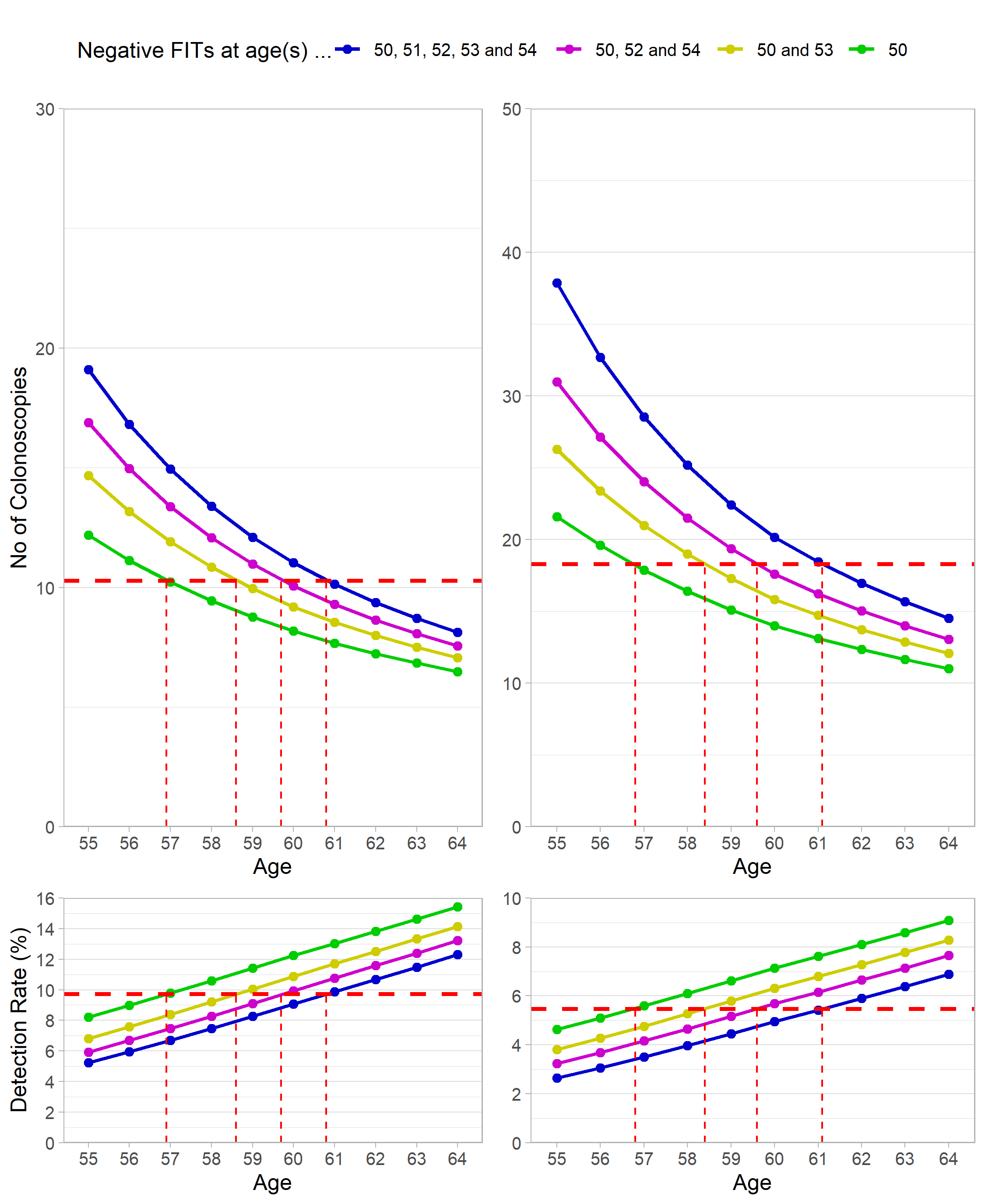 | 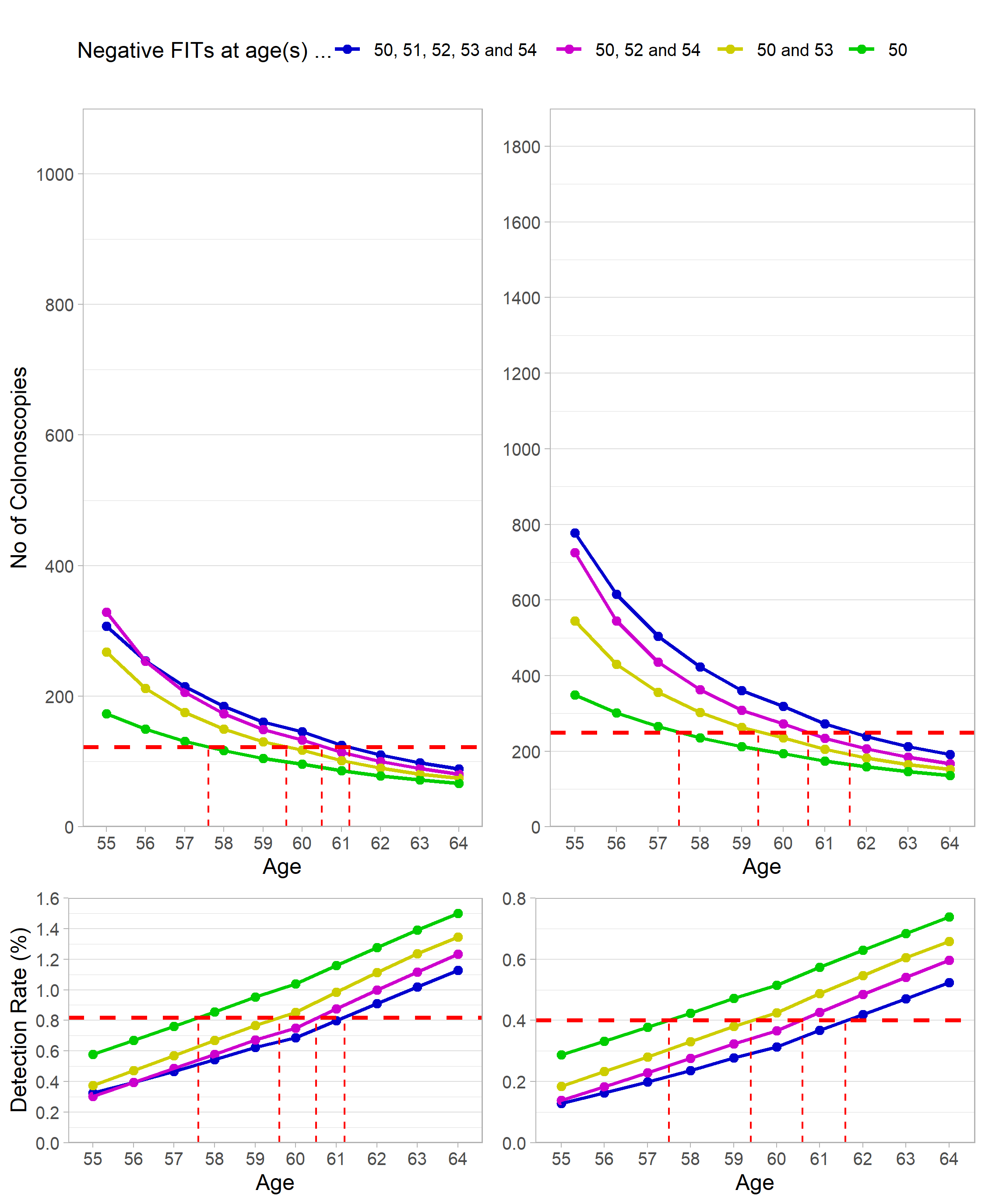 |
