# Supplementary material for: Age-specific sequence of colorectal cancer screening options in Germany: A model-based critical evaluation
Source: PLoS Med. 2020 Jul 17;17(7):e1003194. doi: 10.1371/journal.pmed.1003194 (PMC7367446; doi:10.1371/journal.pmed.1003194)

#### **Supplementary Figure 3** Sensitivity Analysis: PPV for any advanced neoplasm and cancer over 5 rounds of annual FIT testing at ages 50–54.

##### **A1. Starting Prevalences and Transition Rates Lower Limit**


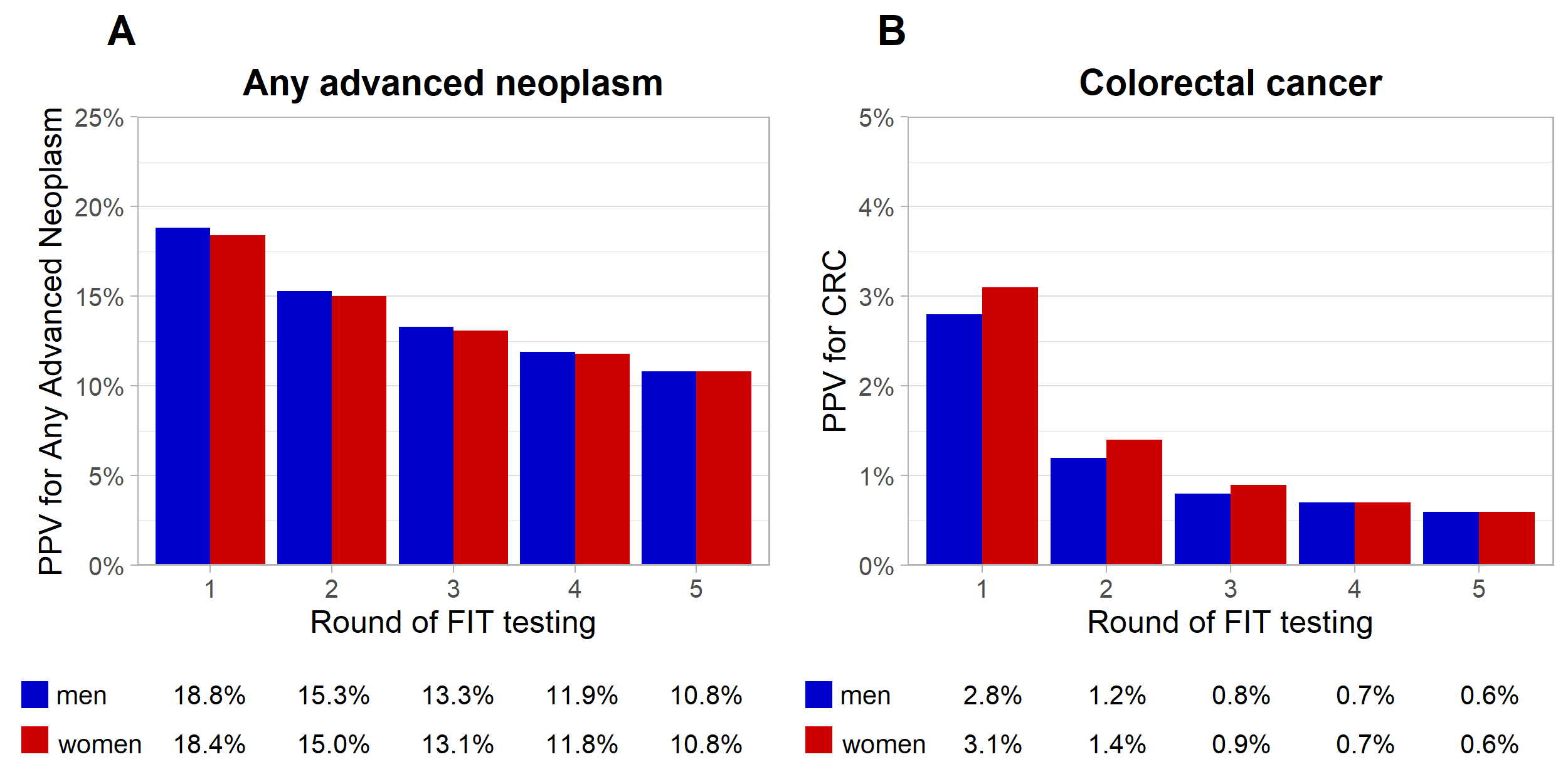


##### **A2. Starting Prevalences and Transition Rates Upper Limit**


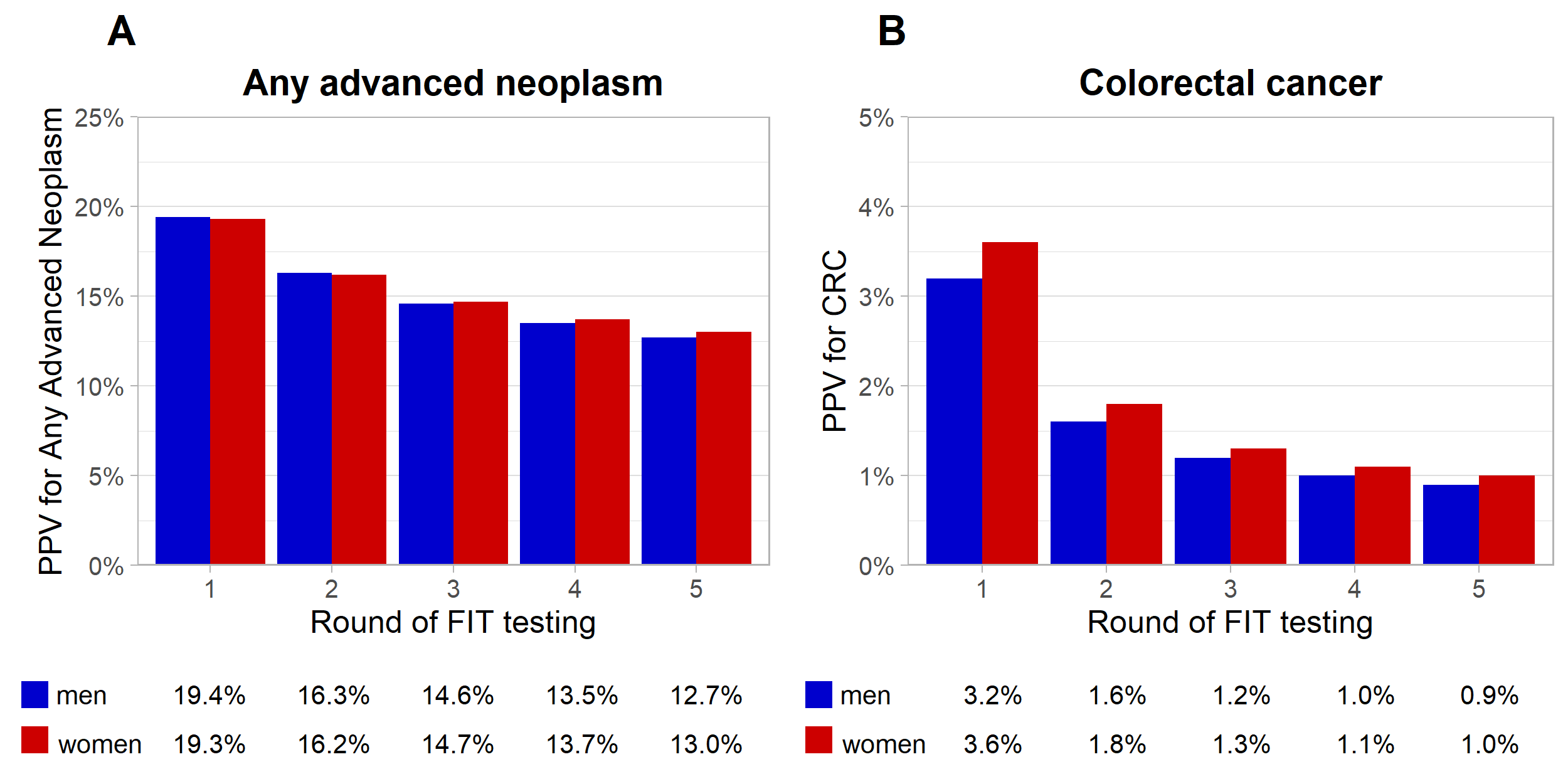


##### **B1. FIT Sensitivity and Specificity Absolute Five Per Cent Points Lower**


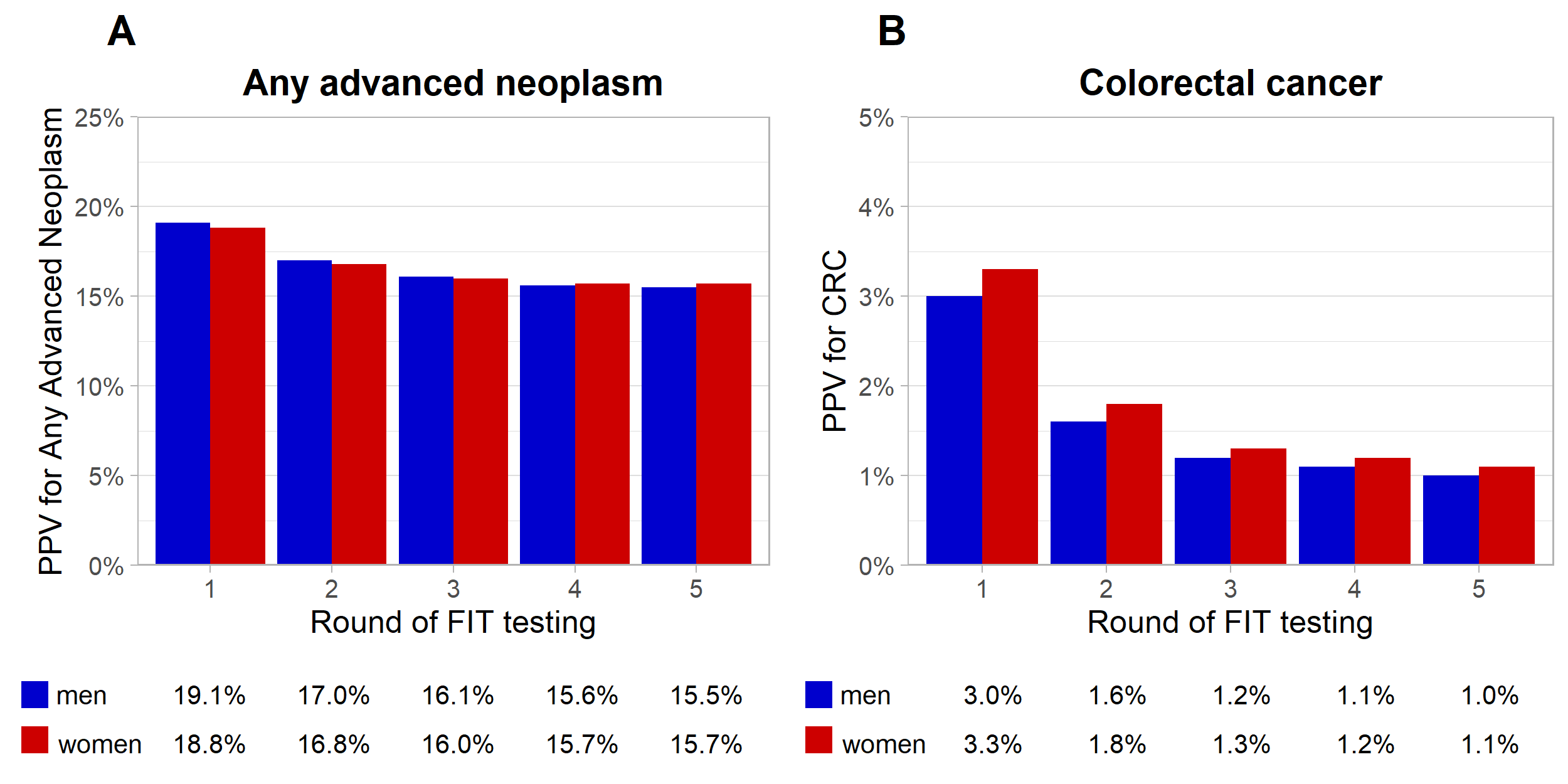


##### **B2. FIT Sensitivity and Specificity Absolute Five Per Cent Points Higher**


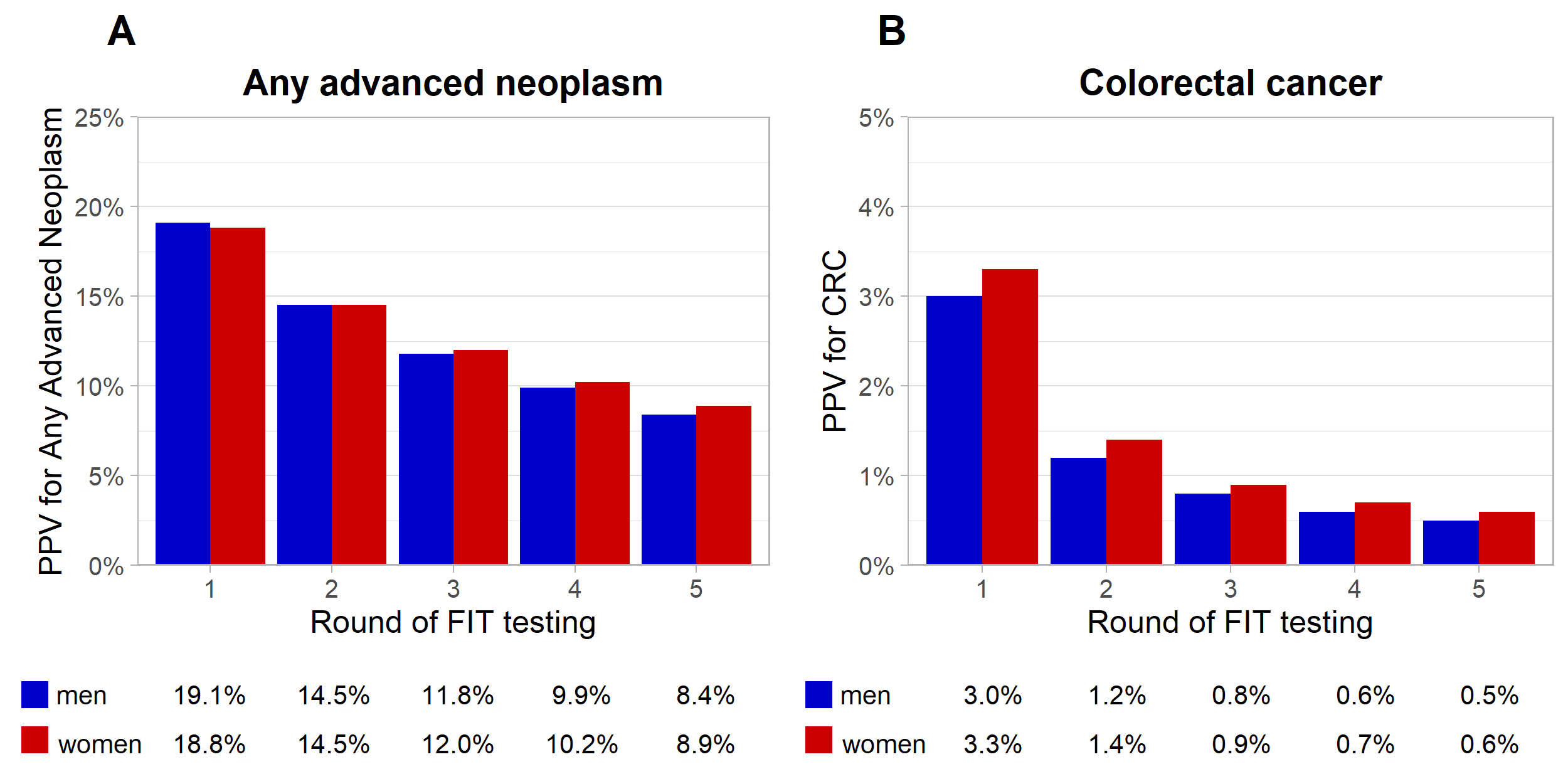


##### **C. Dependencies (Differences In Proneness To Bleed Across Screenees)**


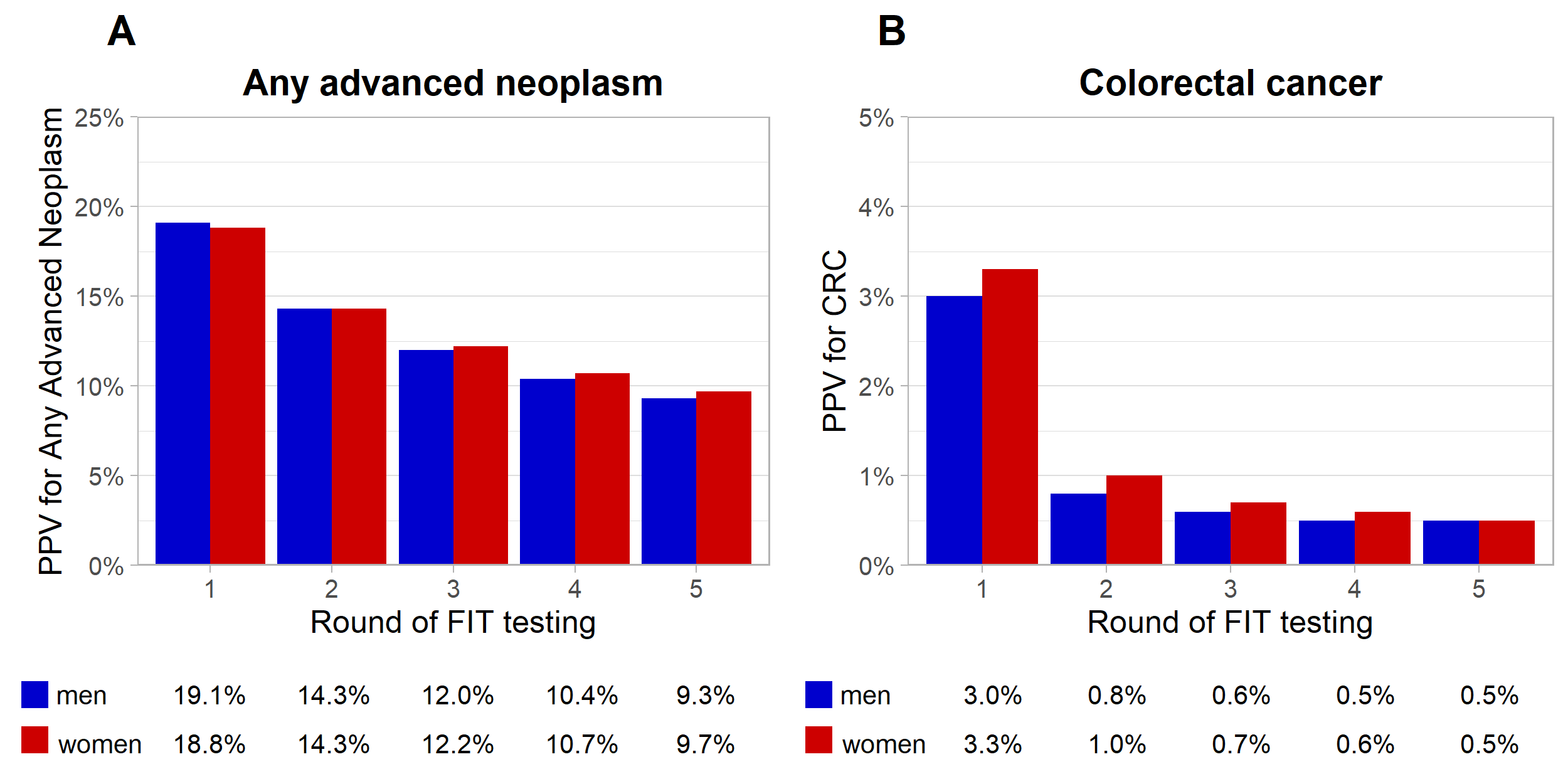

Supplement: S3 Fig — (DOCX) [file pmed.1003194.s004.docx]
