# Supplementary material for: Age-specific sequence of colorectal cancer screening options in Germany: A model-based critical evaluation
Source: PLoS Med. 2020 Jul 17;17(7):e1003194. doi: 10.1371/journal.pmed.1003194 (PMC7367446; doi:10.1371/journal.pmed.1003194)
Supplement: S1 Table — (DOCX) [file pmed.1003194.s005.docx]

#### **Supplementary Table 1** Overview of model parameters

| **A. Proportions of no neoplasm, non-advanced adenoma, advanced adenoma and preclinical CRC at the beginning of simulation^1^** | | | | | |
| --- | --- | --- | --- | --- | --- |
|  |  | **Most advanced finding**  **% (95% confidence interval)** | | | |
| **Sex** |  | **No neoplasm** | **Non-advanced adenoma** | **Advanced adenoma** | **Preclinical colorectal cancer** |
| Men |  | 71.5 (71.3 – 71.7) | 21.7 (21.5 – 21.9) | 6.3 (6.1 – 6.4) | 0.48 (0.45 – 0.52) |
| Women |  | 83.2 (83.0 – 83.3) | 13.2 (13.0 – 13.3) | 3.4 (3.3 – 3.5) | 0.26 (0.24 – 0.29) |
| ^1^ Estimates based on the German screening colonoscopy registry. Extracted and recalculated from reference[1] | | | | | |
|  |  |  |  |  |  |
| **B. Sex- and age-specific annual transition rates between states²** | | | | | |
|  |  | **Annual transition rates**  **% (95% confidence interval)** | | | |
| **Sex** | **Age** | **No neoplasm to non-advanced adenoma** | **Non-advanced adenoma to advanced adenoma** | **Advanced adenoma to preclinical colorectal cancer** | **Preclinical colorectal cancer to clinical colorectal cancer** |
| Men | 50-54 | 3.1 (2.9 – 3.4) | 3.3 (2.8 – 3.9) | 2.6 (2.2 – 3.1) | 17.0 (16.0 – 18.2) |
|  | 55-59 | 3.1 (2.9 – 3.4) | 3.3 (2.8 – 3.9) | 2.6 (2.2 – 3.1) | 17.0 (16.0 – 18.2) |
|  | 60-64 | 3.1 (2.8 – 3.4) | 3.2 (2.6 – 3.7) | 3.1 (2.6 – 3.4) | 18.1 (17.2 – 19.1) |
|  | 65-69 | 3.2 (2.9 – 3.4) | 3.2 (2.6 – 3.7) | 3.8 (3.4 – 4.3) | 20.1 (19.2 – 20.9) |
|  | 70-74 | 2.9 (2.6 – 3.3) | 3.3 (2.6 – 4.0) | 5.1 (4.5 – 5.8) | 19.4 (18.5 – 20.4) |
|  | 75-79 | 2.3 (1.8 – 2.9) | 3.0 (1.9 – 4.2) | 5.2 (4.2 – 6.2) | 19.0 (17.9 – 20.1) |
|  | 80+ | 2.3 (1.8 – 2.9) | 3.0 (1.9 – 4.2) | 5.2 (4.2 – 6.2) | 17.2 (16.0 – 18.8) |
| Women | 50-54 | 1.8 (1.7 – 2.0) | 3.2 (2.6 – 3.8) | 2.5 (2.0 – 2.9) | 20.1 (18.6 – 21.8) |
|  | 55-59 | 1.8 (1.7 – 2.0) | 3.2 (2.6 – 3.8) | 2.5 (2.0 – 2.9) | 20.1 (18.6 – 21.8) |
|  | 60-64 | 2.0 (1.8 – 2.2) | 2.9 (2.2 – 3.4) | 2.7 (2.2 – 3.2) | 21.1 (19.7 – 22.5) |
|  | 65-69 | 2.1 (1.9 – 2.3) | 2.9 (2.3 – 3.5) | 3.8 (3.3 – 4.3) | 20.6 (19.5 – 21.8) |
|  | 70-74 | 2.0 (1.7 – 2.2) | 3.8 (3.0 – 4.6) | 5.0 (4.2 – 5.7) | 19.6 (18.6 – 20.8) |
|  | 75-79 | 1.5 (1.1 – 2.0) | 3.0 (1.7 – 4.4) | 5.6 (4.4 – 6.8) | 18.2 (17.1 – 19.5) |
|  | 80+ | 1.5 (1.1 – 2.0) | 3.0 (1.7 – 4.4) | 5.6 (4.4 – 6.8) | 16.4 (15.3 – 17.8) |
| ² Estimates extracted and recalculated from references [2–4] | | | | | |
|  |  |  |  |  |  |

| **Supplementary Table 1** Overview of model parameters (continued)   \|  \|  \|  \|  \|  \|  \| \| --- \| --- \| --- \| --- \| --- \| --- \| \| **C. Diagnostic performance parameters** \| \| \| \| \| \| \|  \|  \| **Performance (%)** \| \| \| \| \| **Test (sex)** \| **Parameter** \| **No neoplasm** \| **Non-advanced adenoma** \| **Advanced adenoma** \| **Preclinical colorectal cancer** \| \| Colonoscopy (both sexes)**³** \| Sensitivity \| - \| 75.0 \| 95.0 \| 95.0 \| \| Specificity \| 100 \| - \| - \| - \| \| FIT (men)^4^ \| Sensitivity \| - \| 15.7 \| 31.3 \| 80.6 \| \| Specificity \| 91.2 \| - \| - \| - \| \| FIT (women)^4^ \| Sensitivity \| - \| 10.7 \| 26.3 \| 75.6 \| \| Specificity \| 96.2 \| - \| - \| - \| \| ³ Estimates based on references [5,6]  ^4^ Estimates based on references [7,8] and [9–12]. \| \| \| \| \| \| \|  \| \| \| \| \| \| |
| --- | --- | --- | --- | --- | --- | --- | --- | --- | --- | --- | --- | --- | --- | --- | --- | --- | --- | --- | --- | --- | --- | --- | --- | --- | --- | --- | --- | --- | --- | --- | --- | --- | --- | --- | --- | --- | --- | --- | --- | --- | --- | --- | --- | --- | --- | --- | --- | --- | --- | --- | --- | --- | --- | --- | --- | --- | --- | --- | --- | --- | --- | --- | --- | --- | --- | --- | --- | --- | --- |

**References**

1. Brenner H, Kretschmann J, Stock C, Hoffmeister M. Expected long-term impact of screening endoscopy on colorectal cancer incidence: A modelling study. Oncotarget. 2016;7: 48168–48179. doi:10.18632/oncotarget.10178

2. Brenner H, Altenhofen L, Katalinic A, Lansdorp-Vogelaar I, Hoffmeister M. Sojourn Time of Preclinical Colorectal Cancer by Sex and Age: Estimates From the German National Screening Colonoscopy Database. Am J Epidemiol. 2011;174: 1140–1146. doi:10.1093/aje/kwr188

3. Brenner H, Altenhofen L, Stock C, Hoffmeister M. Natural history of colorectal adenomas: birth cohort analysis among 3.6 million participants of screening colonoscopy. Cancer Epidemiol Biomarkers Prev. 2013;22: 1043–51. doi:10.1158/1055-9965.Epi-13-0162

4. Brenner H, Altenhofen L, Stock C, Hoffmeister M. Incidence of colorectal adenomas: birth cohort analysis among 4.3 million participants of screening colonoscopy. Cancer Epidemiol Biomarkers Prev. 2014;23: 1920–7. doi:10.1158/1055-9965.Epi-14-0367

5. van Rijn JC, Reitsma JB, Stoker J, Bossuyt PM, van Deventer SJ, Dekker E. Polyp miss rate determined by tandem colonoscopy: a systematic review. Am J Gastroenterol. 2006;101: 343–50. doi:10.1111/j.1572-0241.2006.00390.x

6. Zhao S, Wang S, Pan P, Xia T, Chang X, Yang X, et al. Magnitude, Risk Factors, and Factors Associated With Adenoma Miss Rate of Tandem Colonoscopy: A Systematic Review and Meta-analysis. Gastroenterology. 2019;156: 1661-1674.e11. doi:10.1053/j.gastro.2019.01.260

7. Gies A, Bhardwaj M, Stock C, Schrotz-King P, Brenner H. Quantitative fecal immunochemical tests for colorectal cancer screening. Int J Cancer. 2018;143: 234–244. doi:10.1002/ijc.31233

8. Gies A, Cuk K, Schrotz-King P, Brenner H. Direct Comparison of Diagnostic Performance of 9 Quantitative Fecal Immunochemical Tests for Colorectal Cancer Screening. Gastroenterology. 2018;154: 93–104. doi:10.1053/j.gastro.2017.09.018

9. Brenner H, Haug U, Hundt S. Sex differences in performance of fecal occult blood testing. Am J Gastroenterol. 2010;105: 2457–2464. doi:10.1038/ajg.2010.301

10. Khalid-de Bakker CAJ, Jonkers DMAE, Sanduleanu S, de Bruïne AP, Meijer GA, Janssen JBMJ, et al. Test performance of immunologic fecal occult blood testing and sigmoidoscopy compared with primary colonoscopy screening for colorectal advanced adenomas. Cancer Prev Res (Phila). 2011;4: 1563–1571. doi:10.1158/1940-6207.CAPR-11-0076

11. Grobbee EJ, Wieten E, Hansen BE, Stoop EM, de Wijkerslooth TR, Lansdorp-Vogelaar I, et al. Fecal immunochemical test-based colorectal cancer screening: The gender dilemma. United European Gastroenterol J. 2017;5: 448–454. doi:10.1177/2050640616659998

12. Brenner H, Qian J, Werner S. Variation of diagnostic performance of fecal immunochemical testing for hemoglobin by sex and age: results from a large screening cohort. Clin Epidemiol. 2018;10: 381–389. doi:10.2147/CLEP.S155548
