# Supplementary material for: Age-specific sequence of colorectal cancer screening options in Germany: A model-based critical evaluation
Source: PLoS Med. 2020 Jul 17;17(7):e1003194. doi: 10.1371/journal.pmed.1003194 (PMC7367446; doi:10.1371/journal.pmed.1003194)
Supplement: S2 Table — (DOCX) [file pmed.1003194.s006.docx]

#### **Supplementary Table 2** Annual CRC-specific mortality rates of CRC patients by mode of cancer detection^1^

|  | **Annual CRC-specific mortality rates (%)** | | | |
| --- | --- | --- | --- | --- |
| **Year after diagnosis** | **Screening colonoscopy– detected cases** | | **Symptom-detected cases** | |
|  | **Men** | **Women** | **Men** | **Women** |
| 1 | 4.6 | 3.7 | 19.7 | 20.6 |
| 2 | 2.2 | 1.9 | 9.3 | 10.7 |
| 3 | 2.1 | 1.3 | 8.8 | 7.4 |
| 4 | 1.5 | 0.9 | 6.3 | 4.8 |
| 5 | 1.2 | 0.6 | 5.0 | 3.3 |
| 6 | 0.8 | 0.3 | 3.5 | 1.7 |
| 7 | 0.4 | 0.3 | 1.8 | 1.8 |
| 8 | 0.4 | 0.3 | 1.9 | 1.8 |
| 9 | 0.4 | 0.0 | 1.9 | 0.0 |
| 10 | 0.0 | 0.0 | 0.0 | 0.0 |

^1^ estimates extracted from references [1,2]

CRC, Colorectal cancer.

**References**

1. Chen C, Stock C, Hoffmeister M, Brenner H. How long does it take until the effects of endoscopic screening on colorectal cancer mortality are fully disclosed?: a Markov model study. Int J Cancer. 2018;143: 2718–2724. doi:10.1002/ijc.31716

2. Chen C, Stock C, Hoffmeister M, Brenner H. Optimal age for screening colonoscopy: a modeling study. Gastrointest Endosc. 2019;89: 1017-1025.e12. doi:10.1016/j.gie.2018.12.021
