# Supplementary material for: Age-specific sequence of colorectal cancer screening options in Germany: A model-based critical evaluation
Source: PLoS Med. 2020 Jul 17;17(7):e1003194. doi: 10.1371/journal.pmed.1003194 (PMC7367446; doi:10.1371/journal.pmed.1003194)
Supplement: S3 Table — (DOCX) [file pmed.1003194.s007.docx]

#### **Supplementary Table 3** Sex- and age-specific general mortality rates

|  | **General mortality rates from age to age +1 (%)**^1^ | |
| --- | --- | --- |
| **Age** | **Men** | **Women** |
| 50 | 0.4 | 0.2 |
| 51 | 0.4 | 0.2 |
| 52 | 0.5 | 0.3 |
| 53 | 0.6 | 0.3 |
| 54 | 0.6 | 0.3 |
| 55 | 0.7 | 0.4 |
| 56 | 0.7 | 0.4 |
| 57 | 0.8 | 0.4 |
| 58 | 0.9 | 0.4 |
| 59 | 1.0 | 0.5 |
| 60 | 1.0 | 0.5 |
| 61 | 1.1 | 0.6 |
| 62 | 1.2 | 0.6 |
| 63 | 1.3 | 0.7 |
| 64 | 1.4 | 0.7 |
| 65 | 1.5 | 0.8 |
| 66 | 1.7 | 0.9 |
| 67 | 1.8 | 0.9 |
| 68 | 1.9 | 1.0 |
| 69 | 2.1 | 1.1 |
| 70 | 2.2 | 1.2 |
| 71 | 2.4 | 1.3 |
| 72 | 2.7 | 1.4 |
| 73 | 3.0 | 1.6 |
| 74 | 3.3 | 1.8 |
| 75 | 3.7 | 2.1 |
| 76 | 4.1 | 2.4 |
| 77 | 4.6 | 2.7 |
| 78 | 5.2 | 3.1 |
| 79 | 5.8 | 3.6 |

*Continued on next page*

**Supplementary Table 3.** *Sex- and age-specific general mortality rates (continued)*

|  | **General mortality rates from age to age +1 (%)**^1^ | |
| --- | --- | --- |
| **Age** | **Men** | **Women** |
| 80 | 6.5 | 4.1 |
| 81 | 7.2 | 4.7 |
| 82 | 8.0 | 5.4 |
| 83 | 8.9 | 6.2 |
| 84 | 9.9 | 7.1 |
| 85 | 11.1 | 8.2 |
| 86 | 12.3 | 9.3 |
| 87 | 13.7 | 10.7 |
| 88 | 15.3 | 12.1 |
| 89 | 16.9 | 13.7 |
| 90 | 18.7 | 15.4 |
| 91 | 20.7 | 17.2 |
| 92 | 22.7 | 19.1 |
| 93 | 24.8 | 21.1 |
| 94 | 27.0 | 23.2 |
| 95 | 29.1 | 25.3 |
| 96 | 31.2 | 27.4 |
| 97 | 33.2 | 29.6 |
| 98 | 35.1 | 31.7 |
| 99 | 37.2 | 34.0 |
| 100 | 39.2 | 36.2 |

^1^Estimates were extracted from German population life tables 2010/2012 (reference[1]).

**References**

1. Rößger F. General Life Table 2010/2012. Methodological Description and Results (Allgemeine Sterbetafel 2010/2012. Methodische Erläuterungen und Ergebnisse). Wiesbaden: Federal Office of Statistics (Statistisches Bundesamt); 2015. Available: www-genesis.destatis.de
