# Supplementary material for: Age-specific sequence of colorectal cancer screening options in Germany: A model-based critical evaluation
Source: PLoS Med. 2020 Jul 17;17(7):e1003194. doi: 10.1371/journal.pmed.1003194 (PMC7367446; doi:10.1371/journal.pmed.1003194)
Supplement: S4 Table — (DOCX) [file pmed.1003194.s008.docx]

#### **Supplementary Table 4** PPV and NPV of 5 consecutive annual FITs at ages 50–54, and numbers of diagnostic colonoscopies after a positive FIT needed to detect 1 case of any advanced neoplasm or 1 CRC, stratified by sex and test round.

| **A. Any advanced neoplasm** | | | | | | | | | | |
| --- | --- | --- | --- | --- | --- | --- | --- | --- | --- | --- |
| **Sex** | **Test round** | **Completed test** | **Subjects with advanced neoplasm** | | **Subjects without advanced neoplasm** | | **PPV** | **NPV** | **Colonoscopy detection rate** | **NNS** |
|  |  | n | TP | FN | FP | TN | % | % | % | n |
| men | 1 | 99,581 | 2,318 | 4,366 | 9,824 | 83,073 | 19.1 | 95.0 | 18.1 | 6 |
|  | 2 | 87,028 | 1,630 | 3,295 | 8,703 | 73,401 | 15.8 | 95.7 | 15.0 | 7 |
|  | 3 | 76,303 | 1,246 | 2,561 | 7,702 | 64,795 | 13.9 | 96.2 | 13.2 | 8 |
|  | 4 | 66,975 | 986 | 2,036 | 6,808 | 57,146 | 12.6 | 96.6 | 12.0 | 8 |
|  | 5 | 58,814 | 798 | 1,651 | 6,011 | 50,354 | 11.7 | 96.8 | 11.1 | 9 |
| women | 1 | 99,770 | 1,083 | 2,557 | 4,663 | 91,467 | 18.8 | 97.3 | 17.9 | 6 |
|  | 2 | 93,778 | 818 | 2,091 | 4,425 | 86,445 | 15.6 | 97.6 | 14.8 | 7 |
|  | 3 | 88,289 | 675 | 1,762 | 4,196 | 81,655 | 13.9 | 97.9 | 13.2 | 8 |
|  | 4 | 83,165 | 579 | 1,520 | 3,975 | 77,092 | 12.7 | 98.1 | 12.1 | 8 |
|  | 5 | 78,353 | 507 | 1,336 | 3,763 | 72,748 | 11.9 | 98.2 | 11.3 | 9 |
|  |  |  |  |  |  |  |  |  |  |  |
| **B. CRC** | | | | | | | | | | |
| **Sex** | **Test round** | **Completed test** | **Subjects with CRC** | | **Subjects without CRC** | | **PPV** | **NPV** | **Colonoscopy detection rate** | **NNS** |
|  |  | n | TP | FN | FP | TN | % | % | % | n |
| men | 1 | 99,581 | 370 | 89 | 11,772 | 87,350 | 3.0 | 99.9 | 2.9 | 35 |
|  | 2 | 87,028 | 145 | 35 | 10,188 | 76,660 | 1.4 | 100.0 | 1.3 | 75 |
|  | 3 | 76,303 | 89 | 21 | 8,859 | 67,334 | 1.0 | 100.0 | 0.9 | 106 |
|  | 4 | 66,975 | 65 | 16 | 7,728 | 59,166 | 0.8 | 100.0 | 0.8 | 126 |
|  | 5 | 58,814 | 51 | 12 | 6,758 | 51,992 | 0.8 | 100.0 | 0.7 | 140 |
| women | 1 | 99,770 | 192 | 62 | 5,553 | 93,962 | 3.3 | 99.9 | 3.2 | 31 |
|  | 2 | 93,778 | 81 | 26 | 5,162 | 88,509 | 1.5 | 100.0 | 1.5 | 68 |
|  | 3 | 88,289 | 52 | 17 | 4,819 | 83,401 | 1.1 | 100.0 | 1.0 | 98 |
|  | 4 | 83,165 | 41 | 13 | 4,512 | 78,598 | 0.9 | 100.0 | 0.9 | 117 |
|  | 5 | 78,353 | 35 | 11 | 4,235 | 74,072 | 0.8 | 100.0 | 0.8 | 130 |

TP: true positive. FN: false negative. FP: false positive. TN: true negative. PPV: positive predictive value. NPV: negative predictive value. NNS: numbers needed to scope
