# Supplementary material for: Age-specific sequence of colorectal cancer screening options in Germany: A model-based critical evaluation
Source: PLoS Med. 2020 Jul 17;17(7):e1003194. doi: 10.1371/journal.pmed.1003194 (PMC7367446; doi:10.1371/journal.pmed.1003194)
Supplement: S5 Table — (DOCX) [file pmed.1003194.s009.docx]

#### **Supplementary Table 5** Sensitivity Analysis: Expected detection rate and associated NNS to detect 1 case of any advanced neoplasm or cancer at screening colonoscopy at age 55 after annual FIT testing from age 50 to age 54, compared to no preceding FIT testing and to strategies with longer intervals between FITs.

##### **A1. Starting Prevalences and Transition Rates Lower Limit**

|  | **Proportion eligible for screening colonoscopy at age 55**^1^ |  | **Any advanced neoplasm** | |  | **Colorectal cancer** | |
| --- | --- | --- | --- | --- | --- | --- | --- |
|  |  |  |  |  |  |  |  |
| **Group** |  |  | **detection rate** | **NNS** |  | **detection rate** | **NNS** |
| **men** |  |  |  |  |  |  |  |
| *no preceding FIT screening* | 97% |  | 9.1% | 11 |  | 0.70% | 143 |
|  |  |  |  |  |  |  |  |
| *Preceding FIT screening*^2^ *…* |  |  |  |  |  |  |  |
| *… annually from age 50 to 54* | 52% |  | 3.4% | 30 |  | 0.08% | 1,312 |
| *… at ages 50, 52 and 54* | 66% |  | 4.9% | 21 |  | 0.13% | 754 |
| *… at ages 50 and 53* | 75% |  | 6.1% | 17 |  | 0.25% | 400 |
| *… at age 50* | 85% |  | 7.6% | 13 |  | 0.48% | 210 |
|  |  |  |  |  |  |  |  |
|  |  |  |  |  |  |  |  |
| **women** |  |  |  |  |  |  |  |
| *no preceding FIT screening* | 98% |  | 5.0% | 20 |  | 0.34% | 298 |
|  |  |  |  |  |  |  |  |
| *Preceding FIT screening*^2^ *…* |  |  |  |  |  |  |  |
| *… annually from age 50 to 54* | 74% |  | 1.9% | 53 |  | 0.04% | 2,499 |
| *… at ages 50, 52 and 54* | 83% |  | 2.7% | 37 |  | 0.07% | 1,433 |
| *… at ages 50 and 53* | 87% |  | 3.4% | 30 |  | 0.13% | 787 |
| *… at age 50* | 93% |  | 4.2% | 24 |  | 0.23% | 432 |
|  |  |  |  |  |  |  |  |

##### **A2. Starting Prevalences and Transition Rates Upper Limit**

|  | **Proportion eligible for screening colonoscopy at age 55**^1^ |  | **Any advanced neoplasm** | |  | **Colorectal cancer** | |
| --- | --- | --- | --- | --- | --- | --- | --- |
|  |  |  |  |  |  |  |  |
| **Group** |  |  | **detection rate** | **NNS** |  | **detection rate** | **NNS** |
| **men** |  |  |  |  |  |  |  |
| *no preceding FIT screening* | 97% |  | 10.4% | 10 |  | 0.94% | 107 |
|  |  |  |  |  |  |  |  |
| *Preceding FIT screening*^2^ *…* |  |  |  |  |  |  |  |
| *… annually from age 50 to 54* | 52% |  | 4.1% | 24 |  | 0.12% | 818 |
| *… at ages 50, 52 and 54* | 66% |  | 5.8% | 17 |  | 0.20% | 488 |
| *… at ages 50 and 53* | 75% |  | 7.2% | 14 |  | 0.38% | 267 |
| *… at age 50* | 85% |  | 8.9% | 11 |  | 0.68% | 147 |
|  |  |  |  |  |  |  |  |
|  |  |  |  |  |  |  |  |
| **women** |  |  |  |  |  |  |  |
| *no preceding FIT screening* | 98% |  | 5.9% | 17 |  | 0.47% | 213 |
|  |  |  |  |  |  |  |  |
| *Preceding FIT screening*^2^ *…* |  |  |  |  |  |  |  |
| *… annually from age 50 to 54* | 74% |  | 2.4% | 42 |  | 0.07% | 1,449 |
| *… at ages 50, 52 and 54* | 82% |  | 3.3% | 30 |  | 0.12% | 869 |
| *… at ages 50 and 53* | 87% |  | 4.1% | 24 |  | 0.20% | 494 |
| *… at age 50* | 93% |  | 5.1% | 20 |  | 0.35% | 287 |
|  |  |  |  |  |  |  |  |

##### **B1. FIT Sensitivity and Specificity Absolute Five Per Cent Points Lower**

|  | **Proportion eligible for screening colonoscopy at age 55**^1^ |  | **Any advanced neoplasm** | |  | **Colorectal cancer** | |
| --- | --- | --- | --- | --- | --- | --- | --- |
|  |  |  |  |  |  |  |  |
| **Group** |  |  | **detection rate** | **NNS** |  | **detection rate** | **NNS** |
| **men** |  |  |  |  |  |  |  |
| *no preceding FIT screening* | 97% |  | 9.7% | 10 |  | 0.82% | 122 |
|  |  |  |  |  |  |  |  |
| *Preceding FIT screening*^2^ *…* |  |  |  |  |  |  |  |
| *… annually from age 50 to 54* | 46% |  | 5.7% | 18 |  | 0.16% | 627 |
| *… at ages 50, 52 and 54* | 62% |  | 6.8% | 15 |  | 0.23% | 433 |
| *… at ages 50 and 53* | 72% |  | 7.8% | 13 |  | 0.38% | 261 |
| *… at age 50* | 83% |  | 8.9% | 11 |  | 0.64% | 157 |
|  |  |  |  |  |  |  |  |
|  |  |  |  |  |  |  |  |
| **women** |  |  |  |  |  |  |  |
| *no preceding FIT screening* | 98% |  | 5.5% | 18 |  | 0.40% | 250 |
|  |  |  |  |  |  |  |  |
| *Preceding FIT screening*^2^ *…* |  |  |  |  |  |  |  |
| *… annually from age 50 to 54* | 62% |  | 3.4% | 30 |  | 0.09% | 1,120 |
| *… at ages 50, 52 and 54* | 75% |  | 4.0% | 25 |  | 0.13% | 788 |
| *… at ages 50 and 53* | 82% |  | 4.5% | 22 |  | 0.20% | 495 |
| *… at age 50* | 90% |  | 5.1% | 20 |  | 0.32% | 314 |
|  |  |  |  |  |  |  |  |

##### **B2. FIT Sensitivity and Specificity Absolute Five Per Cent Points Higher**

|  | **Proportion eligible for screening colonoscopy at age 55**^1^ |  | **Any advanced neoplasm** | |  | **Colorectal cancer** | |
| --- | --- | --- | --- | --- | --- | --- | --- |
|  |  |  |  |  |  |  |  |
| **Group** |  |  | **detection rate** | **NNS** |  | **detection rate** | **NNS** |
| **men** |  |  |  |  |  |  |  |
| *no preceding FIT screening* | 97% |  | 9.7% | 10 |  | 0.82% | 122 |
|  |  |  |  |  |  |  |  |
| *Preceding FIT screening*^2^ *…* |  |  |  |  |  |  |  |
| *… annually from age 50 to 54* | 61% |  | 2.3% | 44 |  | 0.06% | 1,774 |
| *… at ages 50, 52 and 54* | 71% |  | 4.0% | 25 |  | 0.12% | 856 |
| *… at ages 50 and 53* | 78% |  | 5.5% | 18 |  | 0.25% | 405 |
| *… at age 50* | 87% |  | 7.5% | 13 |  | 0.52% | 193 |
|  |  |  |  |  |  |  |  |
|  |  |  |  |  |  |  |  |
| **women** |  |  |  |  |  |  |  |
| *no preceding FIT screening* | 98% |  | 5.5% | 18 |  | 0.40% | 250 |
|  |  |  |  |  |  |  |  |
| *Preceding FIT screening*^2^ *…* |  |  |  |  |  |  |  |
| *… annually from age 50 to 54* | 85% |  | 1.4% | 74 |  | 0.03% | 3,118 |
| *… at ages 50, 52 and 54* | 89% |  | 2.3% | 43 |  | 0.07% | 1,535 |
| *… at ages 50 and 53* | 91% |  | 3.1% | 32 |  | 0.13% | 763 |
| *… at age 50* | 95% |  | 4.3% | 23 |  | 0.26% | 385 |
|  |  |  |  |  |  |  |  |

##### **C. Dependencies (Differences In Proneness To Bleed Across Screenees)**

|  | **Proportion eligible for screening colonoscopy at age 55**^1^ |  | **Any advanced neoplasm** | |  | **Colorectal cancer** | |
| --- | --- | --- | --- | --- | --- | --- | --- |
|  |  |  |  |  |  |  |  |
| **Group** |  |  | **detection rate** | **NNS** |  | **detection rate** | **NNS** |
| **men** |  |  |  |  |  |  |  |
| *no preceding FIT screening* | 97% |  | 9.7% | 10 |  | 0.82% | 122 |
|  |  |  |  |  |  |  |  |
| *Preceding FIT screening^3^ …* |  |  |  |  |  |  |  |
| *… annually from age 50 to 54* | 55% |  | 5.2% | 19 |  | 0.33% | 308 |
| *… at ages 50, 52 and 54* | 67% |  | 5.9% | 17 |  | 0.30% | 329 |
| *… at ages 50 and 53* | 75% |  | 6.8% | 15 |  | 0.37% | 268 |
| *… at age 50* | 85% |  | 8.2% | 12 |  | 0.58% | 173 |
|  |  |  |  |  |  |  |  |
|  |  |  |  |  |  |  |  |
| **women** |  |  |  |  |  |  |  |
| *no preceding FIT screening* | 98% |  | 5.5% | 18 |  | 0.40% | 250 |
|  |  |  |  |  |  |  |  |
| *Preceding FIT screening^3^ …* |  |  |  |  |  |  |  |
| *… annually from age 50 to 54* | 75% |  | 2.6% | 38 |  | 0.13% | 777 |
| *… at ages 50, 52 and 54* | 83% |  | 3.2% | 31 |  | 0.14% | 725 |
| *… at ages 50 and 53* | 87% |  | 3.8% | 26 |  | 0.18% | 545 |
| *… at age 50* | 93% |  | 4.6% | 22 |  | 0.29% | 348 |
|  |  |  |  |  |  |  |  |

*simulated for a hypothetical cohort of each 100,000 men and women.

FIT: fecal immunochemical test. CRC: colorectal cancer. NNS: number of colonoscopies needed to scope one case of any advanced neoplasm or cancer.

^1^ Proportion of the initially simulated 100,000 subjects who are still alive, had no positive FIT, and no diagnosed CRC after all rounds of FIT testing.

^2^ Assumptions: only subjects with negative FIT receive another FIT in the next round. Conditional independence between repeated rounds of FIT testing.

^3^ Assumptions: only subjects with negative FIT receive another FIT in the next round. Subjects with positive FIT receive colonoscopy for diagnostic workup. In this scenario, 20% of subjects never have a positive FIT and 80% of subjects have 25% increased sensitivities and false-positive-rates of FIT testing (i.e. Sensitivity of FIT: non-advanced adenomas, 16.5%; advanced adenomas, 36.0%; preclinical CRC, 97.6%. Specificity FIT: no neoplasm, 92.1%)
